# Serotonin 5-HT_4_ receptor boosts functional maturation of dendritic spines via RhoA-dependent control of F-actin

**DOI:** 10.1038/s42003-020-0791-x

**Published:** 2020-02-14

**Authors:** Yvonne Schill, Monika Bijata, Olga Kopach, Volodymyr Cherkas, Dalia Abdel-Galil, Katrin Böhm, Markus H. Schwab, Michiyuki Matsuda, Valerie Compan, Subhadip Basu, Krystian Bijata, Jakub Wlodarczyk, Lucie Bard, Nicholas Cole, Alexander Dityatev, Andre Zeug, Dmitri A. Rusakov, Evgeni Ponimaskin

**Affiliations:** 1 Cellular Neurophysiology, Hannover Medical School, Carl-Neuberg Str. 1, 30625 Hannover, Germany; 2 Nencki Institute of Experimental Biology, Polish Academy of Sciences, Pasteur Str. 3, 02-093 Warsaw, Poland; 3 UCL Institute of Neurology, University College London, London, WC1N 3BG UK; 4 German Center for Neurodegenerative Diseases (DZNE), Leipziger Str. 44, 39120 Magdeburg, Germany; 5 Bioimaging and Cell Signaling, Kyoto University, Kyoto, 606-8501 Japan; 6 University of Nimes, 30000 Nîmes, France; 7 Computer Science and Engineering, Jadavpur University, Kolkata, 700032 India; 8 Medical Faculty, Otto-von-Guericke-University, Magdeburg, Leipziger Str. 44, 39120 Magdeburg, Germany; 9 Center for Behavioral Brain Sciences (CBBS), Magdeburg, Germany

**Keywords:** Synaptic plasticity, Morphogen signalling, Spine regulation and structure, Cellular neuroscience, Cellular imaging

## Abstract

Activity-dependent remodeling of excitatory connections underpins memory formation in the brain. Serotonin receptors are known to contribute to such remodeling, yet the underlying molecular machinery remains poorly understood. Here, we employ high-resolution time-lapse FRET imaging in neuroblastoma cells and neuronal dendrites to establish that activation of serotonin receptor 5-HT_4_ (5-HT_4_R) rapidly triggers spatially-restricted RhoA activity and G13-mediated phosphorylation of cofilin, thus locally boosting the filamentous actin fraction. In neuroblastoma cells, this leads to cell rounding and neurite retraction. In hippocampal neurons in situ, 5-HT_4_R-mediated RhoA activation triggers maturation of dendritic spines. This is paralleled by RhoA-dependent, transient alterations in cell excitability, as reflected by increased spontaneous synaptic activity, apparent shunting of evoked synaptic responses, and enhanced long-term potentiation of excitatory transmission. The 5-HT_4_R/G13/RhoA signaling thus emerges as a previously unrecognized molecular pathway underpinning use-dependent functional remodeling of excitatory synaptic connections.

## Abstract (web summary)

Schill, Bijata, Kopach et al. show that 5-HT_4_R activation leads to maturation of dendritic spines in hippocampal neurons via G13/RhoA signaling. The dendritic changes are complemented with corresponding changes in excitatory transmission, suggesting a role for 5-HT_4_R in dendritic spine remodeling.

## Introduction

Structural remodeling of excitatory synapses is thought to reflect neural network changes associated with learning and memory formation. The postsynaptic dendritic spines hosting such synapses contain a high concentration of actin, a key element of spine morphogenesis and re-shaping^1–3^. Dynamic changes in the actin cytoskeleton are controlled by small GTPases of the Rho family, including RhoA, Rac1, and Cdc42: Rho GTPases have thus emerged as important regulators of structural plasticity cascades, leading to de novo synapse formation^4,5^. Multiple studies have suggested that Rac1 and Cdc42 promote neurite outgrowth and formation of dendritic filopodia, whereas activation of RhoA triggers neurite retraction^6–8^. However, this dichotomy could be an oversimplification since the ultimate effects of GTPases depend on multiple factors, including their expression level, cellular distribution and the cross-talk between GTPases and their effectors. For example, high activity of Cdc42 can lead to reduced dendritic complexity rather than increased outgrowth^9,10^. Consequentially, defects in Rho-mediated signaling have been suggested to contribute to the development of multiple neurological disorders, including Alzheimer’s disease^11^, schizophrenia^12^ and epilepsy^13,14^. Therefore, if and how Rho-mediated signaling controls remodeling of the excitatory synaptic connections on dendritic spines remains poorly understood.

An established downstream target of small GTPases is the actin-binding protein cofilin. It mediates depolymerization and severance of actin filaments, thus providing new barbed ends for the actin assembly^15–17^. Rho GTPases induce cofilin phosphorylation at serine residue 3 (Ser3), leading to reduced cofilin affinity for actin, which in turn promotes stability and elongation of F-actin^18,19^. In neurons, cofilin is known to play an important role in the structural plasticity of dendritic spines^20^. Although the importance of Rho GTPases in neuronal morphogenesis has been widely accepted, the upstream signaling components of Rho-mediated pathways in neurons have remained enigmatic. We have previously shown that the 5-HT_4_R is coupled to the heterotrimeric G13 protein, which in turn selectively activates the small GTPase RhoA^21^. Recently, post-synaptically expressed 5-HT_4_Rs have been found to gate long-term plasticity of excitatory synapses through a local Ca^2+^-dependent mechanism^22^. This finding has provided initial clues to the long-reported effects of 5-HT_4_R activation on learning, memory, and behavior^23–25^, including pathological changes associated with neurodegeneration^26^. What molecular cascades mediate the action of 5-HT_4_R on synaptic plasticity has, however, remained an important and intriguing question.

Here, we employ high-resolution time-lapse FRET imaging in combination with biochemical approaches to find that the actin-binding protein cofilin acts as a downstream effector of 5-HT_4_R/G13/RhoA signaling, which boosts RhoA activity and increases the local F/G-actin ratio. In neuroblastoma cells, these effects parallel neurite retraction, whereas in hippocampal neurons it triggers F-actin accumulation within dendritic spines leading to the formation of mushroom-type spines. Electrophysiological experiments suggest that 5-HT_4_R activation results in transient cell excitability changes in principal neurons reflected in multi-faceted alterations of excitatory synaptic circuitry.

## Results

### 5-HT_4_R/G13 signaling boosts cofilin phosphorylation

**Fig. 1:**
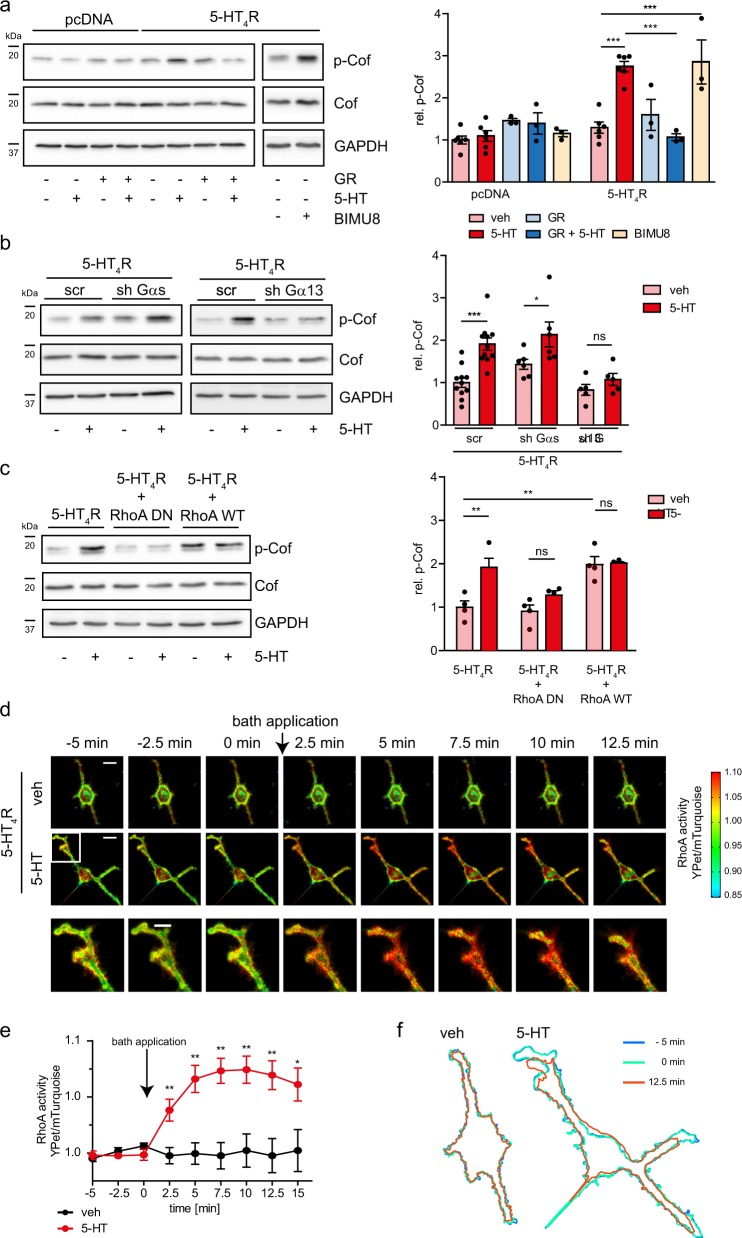
5-HT_4_R activation increases cofilin phosphorylation, RhoA activity, and neurite retraction in the G13-dependent manner. **a** N1E-115 cells transfected with pcDNA or 5-HT_4_R-eGFP were treated for 5 min with 5-HT, BIMU8 alone or in combination with 5-HT_4_R antagonist GR, or vehicle (veh), followed by the detection with antibodies against phosphorylated cofilin (p-Cof), total cofilin (Cof), and GAPDH (bottom) and quantification of the relative cofilin phosphorylation in N1E-115 cells (right). *N* = at least three experiments for each group. See also Supplementary Fig. 1. **b** Representative western blots (left) to visualize cofilin phosphorylation in N1E-115 cells co-transfected with 5-HT_4_R-eGFP and shRNA against Gαs (shGαs) or Gα13 (shGα13), or scrambled shRNA (scr) and treated with 5-HT or vehicle (veh). Quantification of cofilin phosphorylation (right). *N* = 5 experiments for each group. See also Supplementary Fig. 1. **c** Representative western blots (left) to visualize cofilin phosphorylation in N1E-115 cells transfected with 5-HT_4_R-eGFP either alone or in combination with WT or dominant-negative RhoA mutant and treated with 5-HT. Quantification of the relative cofilin phosphorylation (right) in N1E-115 cells (*N* = 4 experiments for each group). See also Supplementary Fig. 1. **d** Time-lapse confocal ratiometric images of the N1E-115 cells co-transfected with 5-HT_4_R-mCherry together with FRET-based biosensor Raichu-RhoA. After 5 min imaging under control conditions, either vehicle or 5-HT was added to the bath solution and cells were imaged for the next 15 min. Increase in the YPet/mTurquoise ratio is equivalent to an increase in the RhoA activity. Scale bar: 20 µm. Lowest row shows the enlargement corresponding to the white box. Scale bar: 10 µm. **e** Quantification of the RhoA activity expressed as a YPet/mTurquoise ratio within the whole cell over time (*N* = 6 cells for each group, at least 3 independent experiments were conducted). See also Supplementary Fig. 2. **f** Contours of cells treated with vehicle (veh) or 5-HT at different time points. See also Supplementary Fig. 2. All data are presented as mean ± SEM. *p < 0.05; **p < 0.01, ***p < 0.001 (two-way ANOVA with Sidak test; **a**–**c**). *p < 0.05, **p < 0.01 (Mann–Whitney test; **e**).

We previously identified the heterotrimeric G-protein G13 and small GTPase RhoA as downstream effectors of 5-HT_4_R^21,27^. Here, we asked whether 5-HT_4_R activation engages these cascades to regulate the phosphorylation status of cofilin. In neuroblastoma N1E-115 cells transfected with 5-HT_4_R, 5-HT application significantly increased cofilin phosphorylation (measured with phospho-cofilin Ser3 antibody; Fig. 1a and Supplementary Fig. 9). This transient effect peaked at 5 min post-stimulation (Fig. 1a and Supplementary Fig. 1a) and was blocked by the high-affinity 5-HT_4_R antagonist GR113808 (GR). The selective 5-HT_4_R agonist BIMU8^23^ increased cofilin phosphorylation to a similar level as did 5-HT in the 5-HT_4_R-expressing cells, but had no effect in the pcDNA transfected cells (Fig. 1a).

In addition to the heterotrimeric G13 protein, 5-HT_4_R is coupled to the stimulatory Gs protein. To determine which G-protein is primarily responsible for cofilin phosphorylation, we developed short hairpin RNAs (shRNAs) to silence endogenously expressed Gαs or Gα13 subunits. Real-time PCR analyses revealed that transfection of N1E-115 cells with the corresponding shRNAs decreased the expression of Gαs and Gα13 mRNAs to 33.3 ± 8.4 and 33.0 ± 9.6% of control, respectively (Supplementary Fig. 1b, d). This was paralleled by a concomitant decrease in the protein expression compared to cells transfected with scramble shRNA (26.8 ± 3.2% of control for Gαs, 58.9 ± 1.5% of control for Gα13; Supplementary Fig. 1c, e). The shRNAs validated this way were then applied to cofilin phosphorylation analysis. The knockdown of endogenously expressed Gα13 protein in 5-HT_4_R expressing N1E-115 cells has effectively canceled the 5-HT_4_R-mediated cofilin phosphorylation boost, while silencing of Gαs subunits had no effect (Fig. 1b and Supplementary Fig. 9). Moreover, we have performed experiments with the membrane-permeant and PDE-resistant PKA inhibitor Rp-8-CPT-cAMPS. Similar to the results obtained after the silencing of Gαs subunits, application of Rp-8-CPT-cAMPS had no effect on the 5-HT_4_R-mediated increase in cofilin phosphorylation (Supplementary Fig. 1f).

We next examined the role of 5-HT_4_R-activated RhoA in regulating cofilin phosphorylation. The 5-HT-evoked increase in cofilin phosphorylation was blocked by the overexpression of a dominant negative (DN) mutant of RhoA (RhoAN19), which acts as a competitive inhibitor of endogenous RhoA activation. Interestingly, the overexpression of wild-type RhoA increased the level of p-cofilin to a value obtained in cells stimulated with 5-HT (Fig. 1c and Supplementary Fig. 9). This effect was comparable with that of 5-HT_4_R-specific agonist BIMU8 (Fig. 1a), confirming that 5-HT_4_Rs are indeed responsible for the signal transduction in these settings. Moreover, pre-treatment of N1E cells with highly selective ROCK inhibitor Y-27632 abolished the 5-HT-evoked increase in cofilin phosphorylation (Supplementary Fig. 3a). Taken together, these results provide evidence that the 5-HT_4_R/G13/RhoA signaling cascade stimulates phosphorylation of the actin-binding protein cofilin in N1E-115 cells.

### 5-HT_4_R stimulation leads to RhoA-mediated neurite retraction

To enable live, high-resolution monitoring of 5-HT_4_R-activated RhoA, we employed a Förster resonance energy transfer (FRET)-based biosensor for RhoA^28^. This biosensor features YPet-tagged RhoA binding domain (RBD) of the protein kinase N (PKN) covalently linked to the mTurquoise-tagged RhoA. Upon activation, conformational changes within the biosensor alter FRET efficiency between acceptor (YPet) and donor (mTurquoise) fluorophores. Because of the 1:1 stoichiometry, RhoA activity can simply be determined by calculation of the acceptor/donor emission ratio. To identify cells expressing both 5-HT_4_R and RhoA sensor, the receptor was C-terminally labeled with mCherry. By combining FRET measurements with time-lapse confocal microscopy, we monitored the spatiotemporal distribution of RhoA activity evoked by 5-HT_4_R stimulation in cell bodies and individual neurites (Fig. 1d, Supplementary Movies 1 and 2). A significant increase in the acceptor/donor ratio (i.e., RhoA activation) was observed already at 2.5 min after agonist application, reaching a maximum between 7.5 and 10 min (Fig. 1e). Notably, the peak of RhoA activation occurred at the tips of neurites; this appeared time-locked with an initiation of neurite retraction (Fig. 1f). In contrast, the treatment of N1E-115 cells co-expressing 5-HT_4_R and RhoA sensor with vehicle (veh) affected neither RhoA activity nor cellular morphology (Fig. 1e, f). Similar results were obtained in cells expressing RhoA sensor alone after treatment with 5-HT (Supplementary Fig. 2a–c). Interestingly, knocking down the endogenous Gα13 with shRNA has effectively canceled the 5-HT_4_R-mediated RhoA activation whereas silencing Gαs subunits had no effect (Supplementary Fig. 1g). Receptor-mediated RhoA activation was highly selective, since the treatment of N1E-115 cells co-expressing 5-HT_4_R and Cdc42 or Rac1 sensors with serotonin affected neither Cdc42 nor Rac1 activity (Supplementary Fig. 2d). These combined findings demonstrate that activation of 5-HT_4_R results in transient and selective RhoA activation, which in turn initiates retraction of neurites.

### 5-HT_4_R/G13 signaling regulates actin cytoskeleton reorganization

**Fig. 2:**
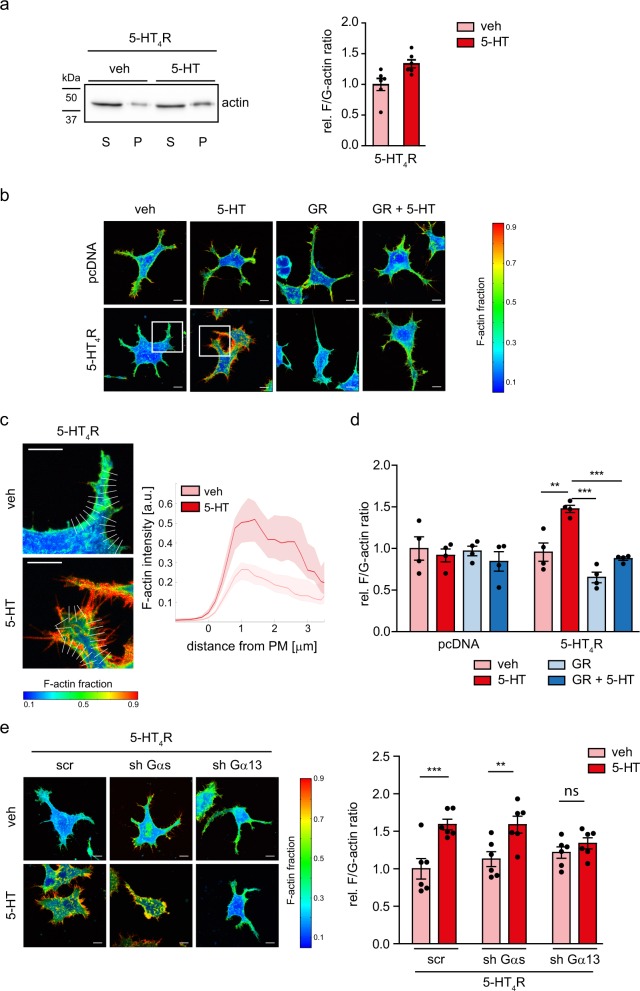
5-HT_4_R activation promotes actin polymerization in N1E-115 cells. **a** Representative western blot (left) from the N1E-115 cells expressing 5-HT_4_R-eGFP after treatment with 5-HT for 10 min, subjected to the ultracentrifugation assay. The pellet fraction (P) contains the F-actin, while the supernatant fraction (S) is highly enriched with the G-actin. Right, the quantification of the relative F/G-actin ratio (*N* = 6 experiments for each group). *p < 0.05 (two-tailed unpaired *t*-test). **b** Confocal images for visualization of the F-actin fraction (F/(F + G)) in the N1E-115 cells expressing 5-HT_4_R-mCerulean treated with 5-HT, 5-HT + GR, and vehicle for 10 min. Scale bar: 10 µm. **c** Enlarged regions of interest (left), selected as white boxes in (**b**) where analysis of the actin polymerization was performed. Array of lines perpendicular to the plasma membrane was automatically generated, along those the analysis of the F-actin staining from outside to inside the cell was determined, Scale bar: 10 µm. Right, quantification of the F-actin staining intensity. **d** Changes in the F/G-actin ratio calculated for the entire cell (*N* = 4 experiments for each group, at least 8 cells analyzed per condition). **p < 0.01, ***p < 0.001 (two-way ANOVA, post hoc Tukey test). **e** Representative confocal images (left) to visualize the F-actin fraction (F/(F + G)) in N1E-115 cells co-transfected with 5-HT_4_R-mCerulean and either scr-shRNA or anti-Gαs shRNA, or anti-Gα13 shRNA and treated with 5-HT or vehicle for 10 min. Scale bar, 10 µm. Right, quantification of the relative changes in the F/G-actin ratio (*N* = 6 experiments for each group with at least 7 cells analyzed per condition). **P < 0.01, ***P < 0.001 (two-way ANOVA, post hoc Sidak test). See also Supplementary Fig. 3. All data are presented as mean ± SEM.

Cofilin plays a key role in stabilization and reorganization of the actin cytoskeleton. To test whether 5-HT_4_R-induced cofilin phosphorylation modulates actin filament dynamics, we first compared the ratio of filamentous and globular actin (F/G-actin ratio) in 5-HT_4_R-expressing N1E-115 cells using an ultracentrifugation approach. 5-HT_4_R activation with 10 µM of 5-HT for 10 min boosted the relative amount of F-actin in the pellet fraction (by 34 ± 7%, *N* = 6, *p* = 0.0194 compared to the supernatant fraction containing G-actin; Fig. 2a and Supplementary Fig. 9).

To understand the intracellular patterns of such effects, we visualized F- and G-actin by staining N1E-115 cells with phalloidin-TRITC and DNaseI-Alexa488, respectively. Based on the ratiometric overlay of phalloidin (F-actin) and DNaseI (G-actin) emissions, we thus mapped the F-actin fraction (F/F + G), which ranges from 0 (low amount of F-actin, high amount of G-actin) to 1 (high amount of F-actin, low amount of G-actin; Fig. 2b). Treatment of 5-HT_4_R-expressing cells with 5-HT robustly increased the F-actin ratio in close proximity to the plasma membrane, with a particular enrichment in neurites and thin protrusions (Fig. 2b). The 5-HT_4_R-mediated F-actin accumulation along the plasma membrane was also revealed by systematically measuring the transmembrane fluorescence intensity profiles (Fig. 2c). The analysis revealed a significant increase in the F/G-actin ratio (by 54 ± 4%, *N* = 4 independent experiments; pcDNA 5-HT, pcDNA GR: 39 cells; 5-HT_4_R GR: 38 cells; pcDNA veh, pcDNA GR + 5-HT, 5-HT_4_R veh, 5-HT_4_R 5-HT, 5-HT_4_R GR + 5-HT: 40 cells; *p* = 0.0038; Fig. 2d). This effect was 5-HT_4_R-specific as it was observed only in the 5-HT_4_R-expressing cells and was completely blocked by the pre-treatment of cells with the receptor antagonist GR (Fig. 2b, d). Moreover, regulation of the F/G-actin ratio could not be explained by the constitutive receptor activity, which was evaluated by treatment of cells with the receptor antagonist GR.

Because 5-HT_4_R can also activate Gαs protein and thus regulate cell morphology, we asked whether Gαs plays a role in the 5-HT_4_R-mediated reorganization of the cytoskeleton. We found that knocking down Gα13, but not Gαs, suppressed the 5-HT-induced increase of the F/G-actin ratio (Fig. 2e), thus ruling out Gαs as a key molecular player here. Therefore, we concluded that activation of the 5-HT_4_R/G13 signaling pathway leads to a pronounced reorganization of the actin cytoskeleton mediated by an increase in the F/G-actin ratio in close proximity of the cell plasma membrane.

To relate the F/G-actin ratio to cell morphology in a quantitative manner, we employed the “compactness” parameter. This parameter reports the ratio between the cell shape area and the polygon area, inscribing it (Supplementary Fig. 3b). Its range from 0 to 1 reflects a transition from branched and elongated to more rounded cells. Treatment of 5-HT_4_R-expressing N1E-115 cells with 5-HT increased the compactness factor, while cells without 5-HT_4_R showed no change upon the respective treatments (Supplementary Fig. 3c). In 5-HT_4_R-expressing cells, in which either Gαs or Gα13 proteins were knocked down by specific shRNAs, cell stimulation with 5-HT increased both compactness and F/G-actin ratio, while knocking down of Gα13 prevented the 5-HT-mediated increase in compactness (Supplementary Fig. 3d). To explore longer-lasting changes in cell compactness, morphology of N1E-115 cells was also evaluated after 90-min stimulation with 5-HT. As a readout, we measured the fraction of rounded cells (since the activation of G13-pathway causes neurite retraction and rounding of neuroblastoma cells^29,30^). The fraction of rounded 5-HT_4_R-expressing cells was increased after treatment with 5-HT, whereas pre-treatment with the receptor antagonist GR blocked this change (Supplementary Fig. 3e, f).

### 5-HT_4_R activation prompts dendritic spine maturation in neurons

**Fig. 3:**
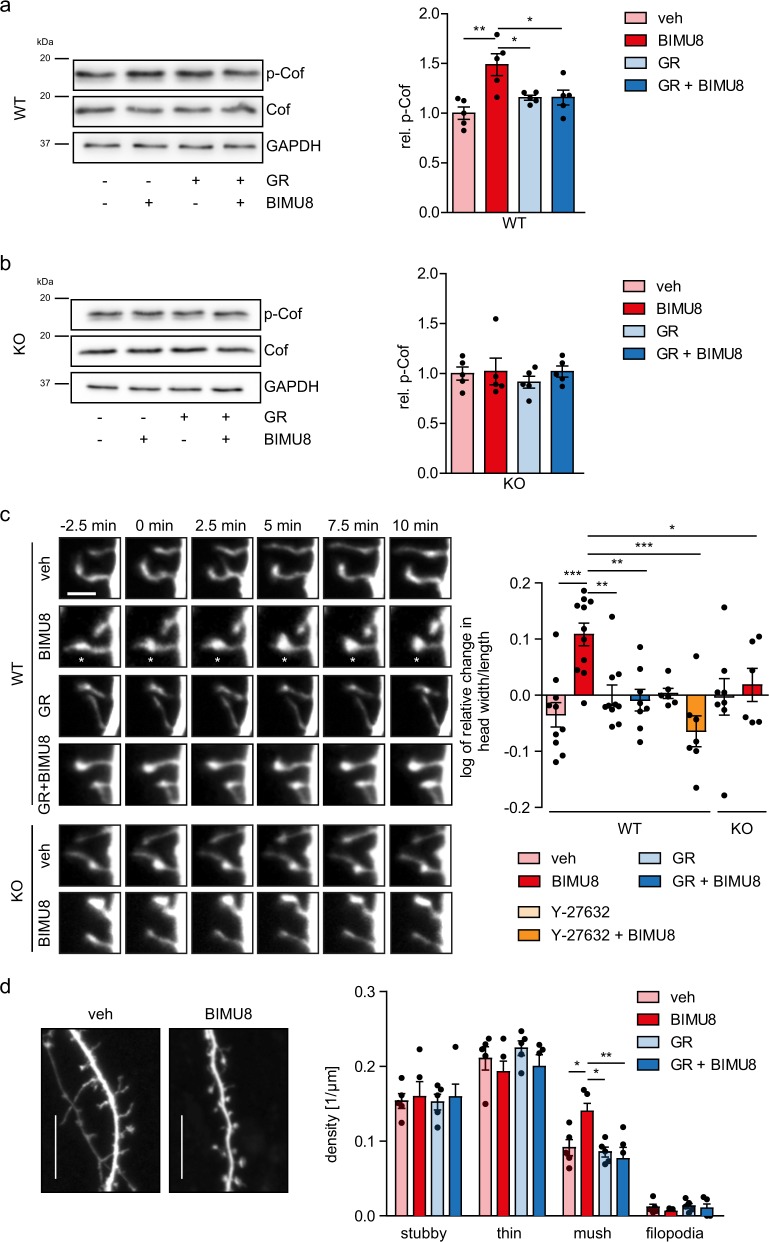
5-HT_4_R activation increases cofilin phosphorylation and induces spine maturation in hippocampal neurons. **a**, **b** Hippocampal neurons (DIV12) isolated from WT (**a**) or 5-HT_4_R-deficient mice (KO) (**b**) were treated with the 5-HT_4_R agonist BIMU8 and/or antagonist GR with or without pre-treatment with the specific ROCK inhibitor Y-27632 (50 µM), followed by the western blot with antibodies against phosphorylated (upper row, p-Cof), total cofilin (middle, Cof) and GAPDH as a loading control (bottom). (Left) Representative western blot showing cofilin phosphorylation in WT neurons (**a**) and in 5-HT_4_R KO neurons (**b**). (Right) Quantification of the relative cofilin phosphorylation in WT neurons (**a**) and in 5-HT_4_R KO neurons (**b**, *N* = 5). **c** Representative time-lapse images (left) of cerulean-expressing hippocampal neurons from WT and 5-HT_4_R KO mice in control (vehicle, veh) and after treatment with BIMU8, GR, GR with BIMU8, Y-27632, and Y-27632 with BIMU8. The asterisk indicates the location of spine that underwent structural plasticity (maturation). Scale bar, 2 μm. (Right) Quantification of changes in the head width/length ratio of dendritic spines at 10 min after treatment of neurons with vehicle (WT: *n* = 425 spines, 10 cells; KO: *n* = 238 spines, 8 cells), BIMU8 (WT: *n* = 435 spines, 11 cells; KO: *n* = 425 spines, 6 cells), GR (WT: *n* = 418 spines, 10 cells), GR with BIMU8 (WT: *n* = 422 spines, 8 cells), Y-27632 (*n* = 248 spines, 6 cells), and Y-27632 with BIMU8 (*n* = 381 spines, 7 cells) *p < 0.05, **p < 0.01, ***p *<* 0.001 (nested ANOVA with Newman–Keuls test). **d** Representative images of secondary dendrites (left) in hippocampal neurons (DIV12) treated for 4 days with vehicle (veh) or BIMU8. Scale bar: 10 μm. (Right) Quantification for different types of dendritic protrusions, including stubby spines, thin spines, mushroom (mush) spines, and filopodia (*N* = 5 cultures, *n* = 90 spines, at least 4 neurons per condition/experiment). *p < 0.05, **p < 0.01 (one-way ANOVA, post hoc Sidak test). See also Supplementary Fig. 4. All data are presented as mean ± SEM.

Cofilin is an important regulator of neuronal morphology and spinogenesis^20,31,32^. To ask whether the 5-HT_4_R-mediated cascade identified in neuroblastoma cells controls cofilin phosphorylation and spine morphogenesis in principal neurons, we explored primary cultures of hippocampal neurons featuring well-established synaptic connections (DIV8-12). The 5-HT_4_R agonist BIMU8 (applied for 10 min at 10 µM) induced a significant increase in cofilin phosphorylation compared to control (veh), which was prevented by the pre-treatment of neurons with the receptor antagonist GR (10 µM) as well as by pre-treatment with the selective ROCK inhibitor Y-27632 (Fig. 3a, Supplementary Figs. 4a and 9). In contrast, BIMU8 had no effect on cultured neurons from the 5-HT_4_R knockout (KO) mice (Fig. 3b and Supplementary Fig. 9), thus confirming a specific involvement of 5-HT_4_R/RhoA signaling.

To test the effect of 5-HT_4_R activation on dendritic spine morphology, we monitored the ‘head width over spine length’ ratio^33^ in individual dendritic spines. The BIMU8 application (10 min, 10 µM) triggered a prominent enlargement of this ratio in all dendritic spines (Fig. 3c). The presence of GR abolished such changes, whereas GR on its own had no effect on spine morphology. Nor did we detect any effect in neuronal cultures from 5-HT_4_R KO mice (Fig. 3c). At the same time, pre-treatment of neurons with Y-27632 abolished BIMU-8 induced changes (Fig. 3c), further confirming the role of 5-HT_4_R/RhoA signaling in the 5-HT_4_R-induced spine maturation.

We also asked whether the 5-HT_4_R activation could induce longer-term morphological changes. We, therefore, incubated the cultures (WT and 5-HT_4_R KO) with a low concentration of BIMU8 (100 nM) and/or GR (2 µM) in bath medium and visualized cell morphology using Cerulean staining. Persistent 5-HT_4_R activation increased the number of mushroom spines only in WT neurons (Fig. 3d), with no effect in 5-HT_4_R KO (Supplementary Fig. 4c). At the same time, the overall spine density and dendritic branching were not affected in either group (Supplementary Fig. 4b, d). These results indicate that 5-HT_4_R signaling plays a role in spine maturation rather than de novo spine formation.

### Multiplexed FRET imaging at individual dendritic spines

**Fig. 4:**
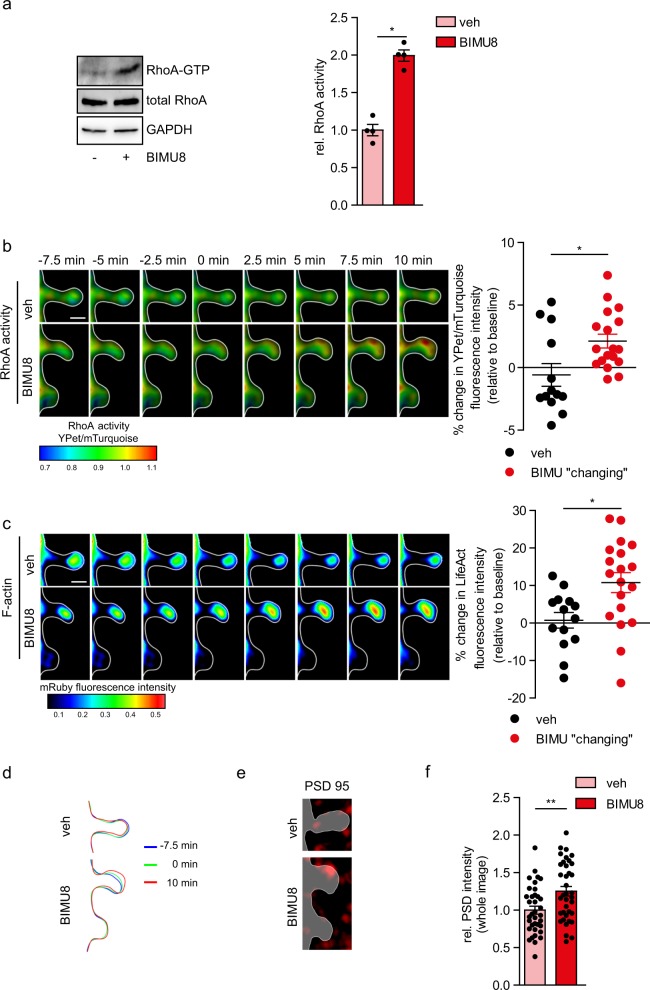
5-HT_4_R activation boosts the RhoA activity and accumulation of F-actin within individual spines. **a** Representative western blot (left) for visualization of active RhoA (RhoA-GTP) (upper), total RhoA (middle), and loading control GAPDH (bottom) in hippocampal neurons (DIV12) treated with BIMU8 or vehicle (veh) for 10 min. Active RhoA was precipitated by pull-down. (Right) Relative RhoA activity calculated as a ratio of RhoA-GTP to total RhoA normalized to GAPDH expression (*N* = 4 cultures for each group). *p < 0.05 (Mann–Whitney *U* test). **b**, **c** Representative time-lapse confocal images of defined spines (left) in the cerulean-expressing hippocampal neurons co-transfected with FRET-based biosensor RaichuRhoA (**b**) and LifeAct-mRuby (**c**). Images were acquired every 2.5 min. After 7.5 min imaging under control conditions (−7.5 min to 0 min), either vehicle or BIMU8 was added to the bath solution and cells were imaged for the further 10 min. Scale bar, 1 µm. Fluorescence intensity for ratiometric changes in the YPet/mTurquoise ratio, reflecting the RhoA activation (**b**) and LifeAct-mRuby, indicating the F-actin accumulation in the same spines (**c**), is shown. (Right) Quantification of the YPet/mTurquoise fluorescence intensity ratio (**b**) and the mRuby fluorescence intensity (**c**) in control (*n* = 13 cells) and BIMU8 responding spines (*n* = 18 cells). *p < 0.05, **p < 0.01 (Mann–Whitney *U* test). See also Supplementary Fig. 5. **d** Spine contours for visualizing morphological changes of dendritic spine in control and BIMU8-treated neurons before (−7.5 and 0 min) and after treatment (10 min). **e**, **f** Post-hoc immunostaining of hippocampal neurons (the same spines shown as in (**b**, **c**) with anti-PSD-95 antibody (**e**) and quantification of relative PSD-95 staining in spines after stimulation with vehicle or BIMU8 (**f**). **p < 0.01 (two-tailed unpaired *t*-test). See also Supplementary Fig. 5. Data are presented as mean ± SEM. In (**b**, **c**), data are presented as relative changes in the YPet/mTurquoise ratio (**b**) and relative changes in F-actin (**c**).

We next tested whether 5-HT_4_R activation increases postsynaptic RhoA activity in hippocampal neurons. First, we used a pull-down with an antibody recognizing an active RhoA and found a significant increase in the level of GTP-bound (i.e., active) RhoA in BIMU8-treated neurons (199 ± 8%, *N* = 4, *p* = 0.028 compared with the vehicle-treated cells; Fig. 4a and Supplementary Fig. 9). To reveal where and when this increase occurs, we set out to monitor, in individual dendritic spines, the real-time RhoA activity (using a RhoA FRET-biosensor) and the F-actin enrichment (using LifeAct-mRuby^34^). BIMU8 application activated RhoA within individual spines within ~2.5 min, reaching peak values between 7.5 and 10 min (Fig. 4b, Supplementary Fig. 5a, and Supplementary Movie 3). Of importance, data from the time series (Fig. 4b, Supplementary Fig. 5a) were corrected ROI-by-ROI for artifacts generated during confocal image acquisition, including bleaching, pixel shift, background, and offset (see “Methods” section for details). The locally corrected sub-regions were then utilized for the quantitative analysis. RhoA activation was paralleled by an increase in the F-actin fraction in the spine head proximity (Fig. 4c, Supplementary Movie 3). Intriguingly, these effects were seen only in spines undergoing morphological changes (i.e., increased spine head width; Fig. 4d). In addition, there was a significant correlation between relative changes in the log width/length of spine and the RhoA activity after BIMU8 treatment (Spearman coefficient of correlation *r* = 0.168, *p* = 0.001). This was not the case for control treatment (*r* = −0.013, *p* = 0.838). Post-hoc immunostaining with 5-HT_4_R antibody and synaptic markers revealed that only 36.4 ± 1.5% of all spines express the 5-HT_4_R (Supplementary Fig. 5b, c): this value is consistent with the proportion of spines affected by BIMU8 application. We also found that in the dendritic spines displaying the 5-HT_4_R-mediated RhoA activation and F-actin enrichment, the PSDs were significantly enlarged (Fig. 4e, f, Supplementary Fig. 5d), in line with spine maturation.

**Fig. 5:**
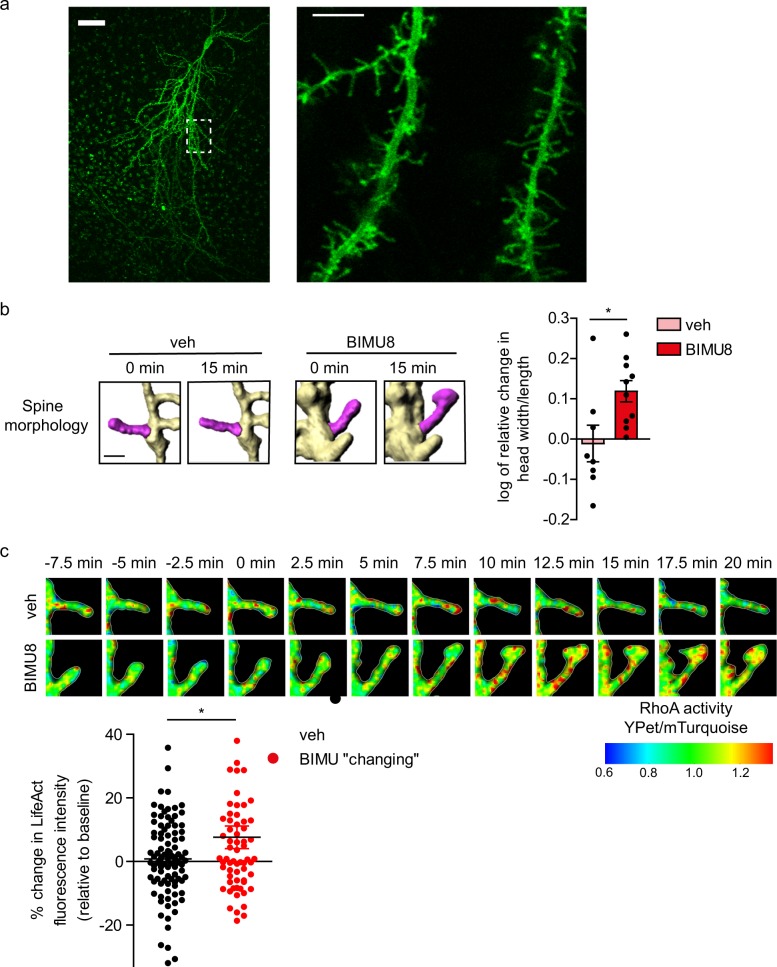
5-HT_4_R/RhoA signaling mediates dendritic spine maturation in the hippocampal tissue. **a** Z-projection image (left) of the hippocampal organotypic tissue (DIV10) with a CA1 pyramidal neuron expressing FRET-based biosensor RaichuRhoA ($\lambda _{{\mathrm{ex}}}^{2{\mathrm{P}}} = 860\,{\mathrm{nm}}$; total scanned 94-μm depth). Dotted rectangle, region of interest for the high-resolution scanning (enlarged on right) for subsequent 3D reconstruction of spines. (Right) A single focal plane image of RaichuRhoA fluorescence (512 × 512 frame image, 0.5-µm Z-step). **b** 3D reconstruction of dendrites from the time-lapse 2P excitation imaging, shown in (**a**), for analysis of morphogenesis in dendritic spines in control tissue (vehicle, veh) and slices following 5-HT_4_R activation with BIMU8 (10 µM, bath application for 20 min). (Right) Quantification of changes in spine morphology, represented as the ratio of the spine head width to the spine length, at 15 min upon stimulation with vehicle (*n* = 156 spines, 8 neurons) or BIMU8 (*n* = 198 spines, 10 neurons). **c** (Upper panel) The time-lapse 2P excitation images of dendritic spines in hippocampal neurons transfected with FRET-based biosensor Raichu-RhoA acquired as Z-stacks every 2.5 min (typically 20–50 optical sections; 512 × 512 pixel frames, 0.5 µm Z-steps, voxel size less than 0.08 µm^3^). After 7.5 min imaging for baseline (RhoA activity under control conditions, −7.5–0 min), either vehicle or BIMU8 was added to the bath solution and the same region of interest was scanned for the next 20 min. Images show the time-course of changes in the RhoA activity within defined spines (color-coded, as indicated on the bottom). (Lower panel) Quantification of the YPet/mTurquoise fluorescence intensity ratio in control and BIMU8-responding spines. *p < 0.05. Data are presented as mean ± SEM. *p < 0.05 (unpaired *t*-test). See also Supplementary Fig. 6.

Importantly, we found a similar pattern of events in organotypic hippocampal slices those largely preserve the architecture of organized tissue^35,36^. To visualize the intracellular RhoA activity in individual neurons within tissue, we used biolistic (gene gun) transfection to express RhoA FRET-biosensor and LifeAct-RFP in sparsely occurring cells (Fig. 5a). High-resolution time-lapse two-photon excitation imaging revealed maturation-type changes in individual spines following BIMU8 application (10 µM, 20 min): at 15 min post-treatment, the spine head width vs length ratio was increased (0.12 ± 0.03, *n* = 198 spines, 10 neurons/slices tested against −0.01 ± 0.04, *n* = 156 spines, 8 neurons/slices from the BIMU8-treated and vehicle groups, respectively; *p* = 0.0197; Fig. 5b, Supplementary Movies 4 and 5). This sequence of events was paralleled by local RhoA activation in spines undergoing morphological maturation (*n* = 10 neurons tested; Fig. 5c and Supplementary Movies 4 and 5). The raw FRET images corresponding to the 3D-reconstructed part of dendrite shown in Fig. 5c are shown in Supplementary Fig. 6. Similar to dissociated cultures, these effects involved no change in the overall spine density and were entirely abolished by the highly potent ROCK inhibitor Y-27632 (50 µM, 1 h of pre-incubation, 57 spines, 5 neurons/slices from the Y-27632-treated group against 36 spines, 3 neurons/slices from the BIMU8-treated group in the presence of Y-27632, *p* = 0.27).

### 5-HT_4_R activation boosts numbers of excitatory synapses

**Fig. 6:**
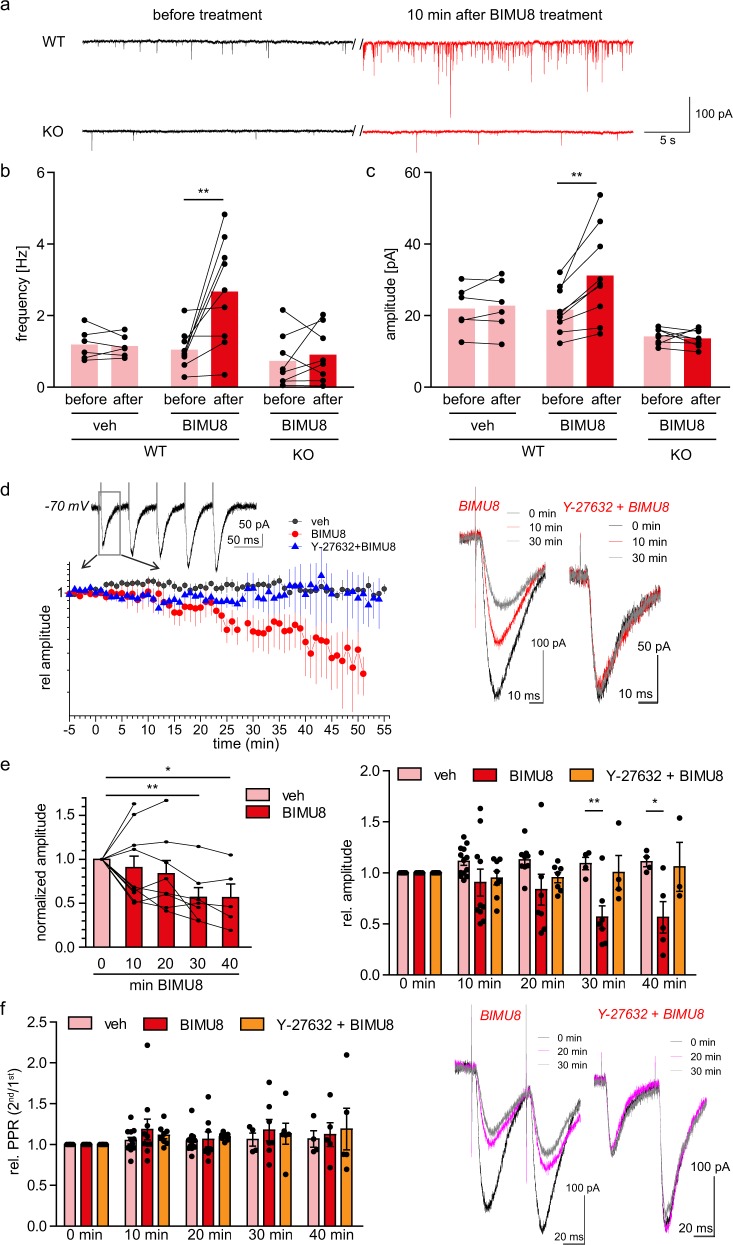
5-HT_4_R activation boosts spontaneous synaptic activity in dissociated cultures while reduces excitatory CA3-CA1 transmission in CA1 pyramidal neurons. **a** Representative recordings of mEPSCs in primary hippocampal neuronal cultures (DIV12) before (black trace) and 10 min after BIMU8 application (red trace) in neurons isolated from WT and 5-HT_4_R-deficient (KO) mice. **b**, **c** Summary of the mEPSC frequency (**b**) and the amplitude (**c**) before and 10 min after BIMU8 application in cultures from WT and KO mice (*n* = 6 cells for vehicle; *n* = 9 cells for BIMU8; *n* = 8 cells for BIMU8 in KO). **p < 0.01 (Wilcoxon paired test). **d** Time course of relative changes in average EPSC amplitude (left) recorded in CA1 pyramidal neurons in response to Schaffer collateral stimulation in control group (veh, *n* = 13 neurons) and slices treated with BIMU8 (10 μM, bath application; *n* = 10 neurons) or with 10 μM BIMU8 in the presence of a ROCK inhibitor, Y-27632 (50 μM, bath application; *n* = 8 neurons). Analysis performed for the first evoked EPSC in a low-frequency pulse train (5 times at 20 Hz, as shown on the top). Right, example traces of evoked EPSCs (first response) at different times after BIMU8 application alone or BIMU8 in the presence of Y-27632, as indicated. **e**. Summary of the relative changes in the EPSC amplitude (first response, as in **d**) at different times after BIMU8 application (left) and statistical comparison between control (veh) and BIMU8-treated groups at different time-points (right) (*p < 0.05, **p < 0.01, unpaired *t*-test). **f** Average paired-pulse ratio (PPR) for the first two EPSCs in CA1 pyramidal neurons evoked by Schaffer collateral stimulation (5 times at 20 Hz, as in **d**) in control (veh) and following BIMU8 application, either alone (red) or in the presence of a ROCK inhibitor Y-27632 (blue), and examples of evoked EPSCs recorded from the same cell at different times after BIMU8 application without or with Y-27632, as indicated. See also Supplementary Figs. 7 and 8. Data are presented as mean ± SEM.

The endogenous ligand of 5-HT_4_R, 5-HT, has been shown to inhibit excitatory transmission in CA1 pyramidal neurons^37^. At the same time, 5-HT_4_R activation boosted evoked excitatory field potentials in CA1 area, with no changes in the afferent input or the transmission efficacy^38^. To understand the underlying mechanisms at the synaptic level, we carried out patch-clamp experiments in dissociated neuronal cultures (DIV12; Fig. 6a) and in CA1 pyramidal neurons in acute hippocampal slices (Supplementary Fig. 7a–d). BIMU8 application (10 min, 10 µM) dramatically increased the average frequency and the amplitude of spontaneous excitatory postsynaptic currents (sEPSCs) in dissociated neuronal cultures, from 1.0 ± 0.2 Hz and 21.6 ± 2.1 pA to 2.7 ± 0.5 Hz (*p* = 0.0078) and 31.2 ± 4.4 pA (*p* = 0.0039), respectively, with no effect of the vehicle (Fig. 6b, c). In the 5-HT_4_R KO, BIMU8 had no effect on the sEPSC frequency or the amplitude (1.2 ± 0.1 Hz, *p* = 0.84; 23 ± 3 pA, *p* = 0.99, Fig. 6a–c). The observed boost in sEPSC could occur because of increases in active synapse numbers or in the network excitability, or both. To understand this further, we recorded miniature (spike-independent) EPSCs (mEPSCs) from CA1 pyramidal neurons in acute slices and found a clear increase in the mEPSC frequency following BIMU8 treatment (from 2.18 ± 0.46 Hz to 3.82 ± 0.54 Hz, *n* = 6 neurons/slices, *p* = 0.0045; Supplementary Fig. 8b). In the meantime, the mEPSC amplitude was not changed significantly in CA1 pyramidal neurons (10.52 ± 0.48 pA vs. 11.48 ± 1.35 pA, *n* = 8242 events recorded for 10 min before BIMU8 treatment against *n* = 12751 events recorded from 6 neurons/slices for the same time 10 min after BIMU8 treatment, *p* = 0.54; Supplementary Fig. 8b). These data suggest that BIMU8 treatment does increase the number of active synapses, albeit without ruling out concurrent increases in cell (or network) excitability.

### 5-HT_4_R activation increases neuronal excitability in the brain

To dissect the physiological effects of 5-HT_4_R activation further, we recorded EPSCs in CA1 pyramidal neurons evoked by Schaffer collateral stimulation in acute hippocampal slices (under GABA transmission blockade). Surprisingly, BIMU8 application (10 µM for 30 min) led to a gradual reduction in the EPSC amplitude, which remained stable in control conditions (Fig. 6d, e). At the same time, the EPSC paired-pulse ratio (PPR, 50 ms interval) remained unchanged in the BIMU8-treated group (Fig. 6f), arguing for the postsynaptic origin of the 5-HT_4_R-dependent changes. The ROCK inhibitor Y-27632 abolished the BIMU8-induced reduction in the amplitude of evoked EPSCs (Fig. 6d, e), with no effect on the PPR ratio (*n* = 8; Fig. 6f). Intriguingly, in the unclamped-cell configuration represented by field excitatory postsynaptic potential (fEPSP) recordings in the stratum radiatum, BIMU8 application (10 µM for 60 min) had a slight facilitating effect (106.8 ± 3.1% of baseline, *n* = 10, vs vehicle application: 100 ± 2.1% of baseline, *n* = 13; *p* = 0.087). The most parsimonious explanation for these observations was that 5-HT_4_R activation, in addition to increasing active synapse numbers, elevates postsynaptic cell excitability: the latter shunts EPSCs (recorded in voltage clamp) while boosting the recruitment of postsynaptic cells in conditions of fEPSP recordings.

Indeed, we found that application of BIMU8 could induce significant inward current, hence membrane depolarization in recorded cells, sometimes followed by hyperpolarization (Supplementary Fig. 8c–e). Thus, in addition to synaptic changes, 5-HT_4_R activation can elevate intrinsic excitability of postsynaptic cells. Consistent with these effects, BIMU8 treatment enhanced the level of long-term potentiation (LTP) recorded 50–60 min after the theta-burst stimulation (fEPSP changes were 142.7 ± 2.6%, *n* = 13 slices in control vs. 157.9 ± 4.3 %, *n* = 10 after BIMU8, *p* = 0.004; Supplementary Fig. 7a, b). The synaptic potentiation induced by 5-HT_4_R activation is consistent with other reports^39^ and was eliminated by Y-27632 (Supplementary Fig. 7c, d). At the same time, BIMU8 treatment had no effect on the short-term synaptic potentiation in the absence or presence of Y-27632 (Supplementary Fig. 7b, d).

## Discussion

In the CNS, coordinated assembly and disassembly of the actin cytoskeleton underpins neurite outgrowth, dendrite formation, and the development and use-dependent plasticity of the dendritic spines hosting excitatory synapses^40,41^. This process is regulated by small GTPases of the Rho family, some which were previously shown to undergo selective activation by the 5-HT4R. The overall aim of the present study was, therefore, to understand whether and how 5-HT_4_R activation controls remodeling of the excitatory synaptic connections on dendritic spines.

First, we used an experimental model of neuroblastoma cells to find that 5-HT_4_R activation boosts phosphorylation of cofilin, which facilitates formation of the filamentous F-actin^15,42–45^. However, upstream of cofilin phosphorylation, 5-HT_4_R couples to two different heterotrimeric G-proteins, Gαs and Gα13^21,46^, each of which activates different signaling pathways^30,47–50^. In particular, Gs-mediated changes in cAMP levels are known to exert diverse effects on the cytoskeleton remodeling via effectors of cAMP, like PKA and Epac^51,52^. We, therefore, developed short hairpin RNAs (shRNAs), enabling selective silencing of endogenous Gαs or Gα13, and also applied the PDE-resistant PKA inhibitor Rp-8-CPT-cAMPS, to show that the 5-HT_4_R-mediated cofilin phosphorylation and its downstream effects are mediated solely by the Gα13 protein. We employed high-resolution live FRET imaging inside targeted cells to confirm that RhoA acts as an intermediate effector connecting the 5-HT_4_R/Gα13 signaling complex to cofilin-mediated changes in the actin cytoskeleton reorganization.

The 5-HT_4_R/G13/RhoA signaling cascade also occurs in neurons. Indeed, cofilin activity is key to dendritic spine remodeling^53,54^ and its increase has been associated with use-dependent spine shrinkage, whereas a phosphorylation-prompted decrease in cofilin activity has been related to the ‘maturation-type’ spine enlargement^20,55,56^. Correspondingly, bi-directional changes in cofilin activity are involved in synaptic potentiation during chemical LTP^55^. We documented the 5-HT_4_R-dependent cofilin phosphorylation in hippocampal neurons which was then associated with dynamics of RhoA activity, increase of F-actin fraction, and changes in dendritic spine morphology. The results thus provided a causal link between the 5-HT_4_R activation and dendritic spine remodeling (maturation-type) in central neurons. The real-time multiplex FRET imaging revealed the 5-HT_4_R-mediated activation of RhoA at close proximity to the spine head, followed by a pronounced accumulation of filamentous actin and, eventually, spine enlargement.

Other morphological traits of the studied nerve cells with well-formed synaptic connections (DIV12 and older), such as the length and the number of neurites, the complexity of dendritic branching and spine density were not affected by 5-HT_4_R. In contrast, our previous study in immature neurons (DIV1) reported that 5-HT_4_R activation reduced the number of neurites and decreased the neurite length, the effects comparable to those in neuroblastoma cells^27^. Thus, the morphogenetic effects of 5-HT_4_R activation in the developed synaptic network appear limited to synaptic connections. One parsimonious explanation is that the expression profiles of downstream effectors, in particular the Rho GTPase signaling network, change during cell maturation from a cell-spread type to a spine-concentrated type. Current models for Rho GTPases assume that Rac1 and Cdc42 regulate neurite outgrowth, while RhoA controls neurite retraction^6,7,57^. However, this simplified view is not consistent with the finding that multiple GEFs, GAPs, and effectors outnumber their cognate Rho GTPases^58–60^, suggesting a more complex scenario in which multiple spatiotemporal Rho GTPase signaling networks (including GAP, GEFs, GTPases, and effectors) modulate different morphological processes. A recent observation that different RhoA-specific GAPs regulate two distinct RhoA signaling complexes lends support to this hypothesis: while ARHGAP5 is involved in the RhoA-mediated growth cone collapse, RhoA/DLC1 might trigger mDia1 formin to polymerize F-actin and to enable filopodia extension^61^. Whether the expression profile of RhoA-relevant GAPs and GEFs is indeed modulated during postnatal development thus remains an important and open question.

We have recently reported that a distinct serotonin receptor type, 5-HT_7_R, prompts morphological effects opposite to those reported here for the 5-HT_4_R: activation of the 5-HT_7_R facilitates neurite outgrowth, initiates the formation of new synapses and leads to elongation of dendritic spines via the G12-dependent activation of Cdc42^27,33,39,62^. We also found that expression levels of 5-HT_4_R and 5-HT_7_R in the hippocampus are differentially regulated during postnatal development. While the expression of 5-HT_4_R remains constant during the early postnatal phase and slightly increases at postnatal day 90 (P90), 5-HT_7_R is strongly expressed only during early postnatal stages (P2 and P6) and is dramatically downregulated during later development (P21)^39^. These data suggest that an interplay between 5-HT_4_R- and 5-HT_7_R-mediated signaling may represent a mechanism by which serotonin differentially modulates neuronal morphology during development: in the early postnatal stages, 5-HT_7_R-mediated signaling is mainly responsible for arborization of dendritic trees, spinogenesis, and formation of basal neuronal connections, while at the later developmental stages, activation of the 5-HT4R is involved in maturation and stabilization of spines.

The physiological consequences of 5-HT_4_R activation for synaptic circuit function appear two-fold. First, the receptor activation boosts maturation of synaptic connections, thus increasing the number of active axo-spinous excitatory synapses in dendritic branches of principal neurons. The present study unveils the key molecular mechanisms acting inside dendritic spines to underpin such effects. Second, 5-HT_4_R activation can have a significant, if transient, boosting effect on cell excitability, consistent with a tonic depolarizing current evoked by a 5-HT_4_R agonist. This effect can partly shunt evoked synaptic responses (reflected in a decrease of the amplitudes of evoked EPSCs) while boosting excitatory network activity (reflected in an increase of the amplitudes of spontaneous EPSCs and fEPSPs). We observed a dramatic (2–3-fold) decrease in the evoked EPSCs by 5-HT_4_R activation, with no such effects on miniature events. Given that the NMDAR component usually peaks only at 15–20% of the EPSC amplitude at these synapses, the AMPA/NMDAR ratio change cannot explain the paradoxical discrepancy between miniature and evoked EPSC recordings. Similarly, these effects cannot be explained by changes in the AMPA receptor desensitization because its influence transpires at relatively high release frequencies and high occupancy of AMPA receptors.

The excitability effects reported here appear consistent with several observations reported previously. 5-HT_4_R were shown to modulate several types of K^+^-channels^22,63^ that leads to a long-lasting increase in neuronal excitability, and a previous study in adult rats showed that activation of 5-HT_4_R converted weak synaptic potentiation into persistent LTP in the CA1 area^64^. It has also been shown that 5-HT_4_R activation could prevent the learning-induced facilitation of LTD^65,66^ and depotentiation of LTP, in both CA1 and dentate gyrus^67^, while regulating LTD in other brain regions through the modulation of the postsynaptic BK channels^22^. Thus, electrophysiology has suggested that, in addition to the synaptic plasticity effects revealed by the molecular biology exploration, 5-HT_4_ activation might also affect cell and/or network excitability. The latter effect does not detract from the importance of findings pertaining to synaptic plasticity (the main focus on the present study), but suggests that the ultimate consequences for brain circuitry could be complex and multi-faceted.

The present data also provide insights into the molecular machinery underpinning the 5-HT_4_R-dependent dendritic spine growth after learning in rodents^68^, which seems to augment learning and memory in rodents^24,69–72^ and primates^73^. Clearly, while the present study provides detailed evidence for the molecular machinery underpinning synaptic changes prompted by 5-HT_4_R activation, the precise mechanisms pertinent to the 5-HT_4_R-dependent changes in cell excitability remain to be ascertained.

## Methods

### Animals

All procedures performed on animals were according to the guidelines of the European Commission (European Communities Council Directive 2010/63/EU) and the United Kingdom Home Office (Scientific Procedures) Act (1986). The following mouse strains were used in this study: C57BL6/J and the B6;129SvEv-Htr4 mice (WT+/+, KO−/−).

### Cell lines

N1E-115 cells were grown in Dulbecco’s modified Eagle’s medium (DMEM) containing 10% fetal calf serum and 1% penicillin/streptomycin at 37 °C under 5% CO_2_. For transient transfection, cells were seeded at low-density on 35 mm dishes or on poly-L-Lysin coated 18 mm coverslips for biochemical or microscopic analysis, respectively, and were transfected with corresponding plasmids using Lipofectamine 2000 Reagent (Invitrogen). Cells were cultured 24 h post-transfection in a serum-free DMEM to induce morphological differentiation.

### Primary hippocampal cultures

Hippocampal cultures were prepared from C57BL6/J mice and from the B6;129SvEv-Htr4 mice (WT+/+, KO−/−) at postnatal day 0–1 (P0–P1) according to an optimized protocol^39,74^. Briefly, hippocampi were isolated and dissociated neurons were plated at a density of 25–30 × 10^3^ cells per coverslip onto sterilized 18 mm coverslips coated with 250 µg/ml PLL and 50 µg/ml PDL. After 1 h incubation at 37 °C, Neurobasal-A medium (Gibco) containing 2% (v/v) B27 supplement, 1% (v/v) GlutaMAX® and 0.2% (v/v) penicillin–streptomycin was added. Cultures were maintained at 37 °C/5% CO_2_. Cells were transfected using Lipofectamine 2000 Reagent (Invitrogen) according to the manufacturer’s protocol at DIV7. On DIV12, neuronal cultures were subjected to analysis.

### Organotypic hippocampal tissue

Hippocampal slice cultures were prepared from the Sprague-Dawley rat pups (P6–P8 day-old) according to ref. ^75^. Briefly, hippocampi were isolated, transverse slices were cut (350 μm thick) and placed on 0.4-μm membrane inserts (Millicell®CM, Millipore). Slices were maintained in culturing medium containing MEM supplemented with 25% HBSS, 25% horse serum, 28 mM glucose, 1 mM GlutaMax, Penicillin–Streptomycin (100 U/ml) at 35 °C in 95% O_2_ and 5% CO_2_. Medium was replaced every 2–3 days. Transduction of cells with RhoA sensor (35 µg DNA) and LifeAct-RFP (15 µg DNA) plasmids was performed at 5 DIV, using gene gun approach with gold microcarriers (6.25 mg, 1.6 µm diameter, Bio-Rad, UK) in the presence of 5 µM of Ara-C under a 120-psi pressure.

### Recombinant DNA

RhoA-WT or RhoA-DN (N19) mutant were provided by Dr. A. Woehler (MDC Berlin, Germany). The pmRFPruby-N1-Lifeact plasmid was obtained from Peter Claus (MHH, Hannover, Germany) and FRET-based RhoA biosensor pPBbsr2-RaichuEV-RhoA-KRasCT was reported previously (Komatsu et al.^28^). pTracer CMV2 vector encoding 5-HT_4_R was described previously^21^. The 5-HT_4_R sequence was cloned into a pcDNA 3.1 vector and fused C-terminally to an eGFP, mCherry or mCerulean. Plasmid encoding for Cerulean under control of synapsin promotor was cloned into pDBR in which the CMV promotor was replaced by hSyn promotor. The short hairpin RNAs (shRNAs) against Gαs and Gα13 were created by Oligoengine into a pSUPER.neo + GFP and a pSUPERIOR vector. The target sequences for these shRNA constructs were 5′-CCCCAACCAGACTAACCGCCTGTTCAAGAGACAGGCGGTTAGTCTGGTTGTTTT-3′ for Gαs and 5′-CCCGTGTTCCTGCAGTATCTTCTTCAAGAGAGAAGATACTGCAGGAACACTTTT-3′ for Gα13. A previously described scramble (scr) shRNA^39^ was used as a control.

### Analysis of cofilin phosphorylation

Agonist-induced changes in cofilin phosphorylation were analyzed by western blotting using polyclonal anti-cofilin (Cell Signaling Technology) and polyclonal anti-phospho-Cofilin (Ser3; Santa Cruz Biotechnology) antibodies. Relative cofilin phosphorylation was calculated as a ratio of p-Cof to total Cof normalized to GAPDH expression.

### Real time (RT) quantitative PCR

The efficiency of different shRNAs against Gαs and Gα13 was determined by RT-qPCR with specific TaqMan® probes against target DNA (Applied Biosystems). For reasons of clarity and comprehensibility, we showed only the best working shRNA from three different tested shRNAs. For RT-qPCR, mRNA was isolated from N1E-115 cells transfected either with control shRNA or with the different shRNA constructs against Gαs and Gα13. For the detection of Gαs and Gα13 mRNA, corresponding Gene Expression Assays (Applied Biosystems) containing gene-specific primers and FAM-probes were used. For quantitative analysis, eukaryotic GAPDH RNA was analyzed in parallel. The analysis was performed by using delta-delta-Ct method as described previously^39^.

### Measuring the F/G-actin ratio and fraction

Amount of the F- and G-actin in lysed N1E-115 was determined accordingly to the method described elsewhere^76^. After ultracentrifugation, equal amounts of F- and G-actin containing lysates were loaded on a SDS gel and quantified after the western blot analysis using an anti-α-actin antibody. In order to determine the F/G-actin ratio at the single-cell levels, fixed N1E-115 cells were stained with phalloidin-TRITC (Sigma-Aldrich) to visualize F-actin and with DNase I-Alexa488 (Life Technologies) to visualize G-actin, using a Zeiss LSM 780 with a 63x oil immersion objective and 1.4-fold zoom. Images were acquired in online fingerprinting mode. Data were analyzed using custom-written Matlab scripts by applying the following schema: correction for background, scaling to the 99.9% percentile intensity of the control condition of each experiment, and thresholding using unimodal background-symmetry method. Finally, z-maximum projection was calculated from averaged actin intensity. For visualization and for statistical analysis, the voxel-based F-actin fraction *FR*_F-actin_ = *I*_F-actin_/(*I*_F-actin_ + *I*_G-actin_) and ratio *R* = *I*_F-actin_/*I*_G-actin_ were calculated. A gamma value of 0.8 was applied to the images to enhance the visualization of small structures. To determine the actin distribution close to the plasma membrane, lines perpendicular to the cell surface were drawn and intensity profiles were calculated as described^77^.

### Morphological analysis of N1E-115 cells

Changes in N1E-115 cell shape were monitored in a blinded fashion using the Zeiss LSM780 confocal microscope. Cells were either divided in rounded, flattened, or neurite-bearing ones^21^. For each experiment, the fraction of rounded, flattened and neurite-bearing cells was calculated from 100 to 400 cells, and morphologies were scored in a blinded fashion.

### Immunocytochemistry and morphometric analysis

Neuronal morphology was visualized by transfection with a plasmid encoding for Cerulean. Images were acquired using a Zeiss LSM 780 confocal microscope (LD C-Apochromat 40×/1.1 W, excitation: 440 nm, z-stacks with 1024 × 1024 pixel, voxel size: 0.07 × 0.07 × 0.35 µm). Density of dendritic protrusion and changes in spine shape were analyzed in 2D mode using a custom-written software (SpineMagick software)^78^. Dendritic spine morphometry was analyzed quantitatively using the spine head width/spine length ratio as a scale-free shape parameter as recently described^33^ and by spine classification into different spine types. Here, protrusions longer than 4 μm were defined as dendritic filopodia, while those with a length to neck width ratio smaller than 2 were defined as stubby spines^79^. The remaining spines were classified into mushroom spines (spine head width >0.75 μm) and thin spines^80,81^. To minimize variation in the spine head width/spine length ratio resulting from the diversity of spines and the spontaneous fluctuations of the spine shape, the same spines were identified in the time-series of images and the relative changes (i.e., x_1_/x_0_) were plotted in log scale.

Sholl analysis of hippocampal neurons was performed using the software Fiji and its plugin NeuronJ.

For 3D morphometric analysis in organotypic slices, we applied a method for 3D segmentation of dendritic spines using a multi-scale opening approach^82^. The method estimates 3D morphological attributes of individual dendritic spines for the effective assessment of their structural plasticity. It uses basic mathematical notations to define different key spine compartments (e.g., spine head and spine neck) and experimentally verified that the quantitative analysis of the defined spine attributes accurately models spine plasticity. The approach allows the user to mark specific dendritic spines, segment the spines as 3D volumes, and extract relevant morphometric features with high accuracy and minimal user intervention. The method was used to precisely describe the morphology of individual spines in real-time using consecutive images of the same dendritic fragment.

For the analysis of PSD-95 distribution in dissociated hippocampal neurons, fixed neurons were stained with mouse monoclonal primary anti-PSD-95 antibody followed by incubation with donkey anti-mouse DyLight 649 conjugated secondary antibody. Image analysis was performed on Zeiss LSM 780 confocal microscope with a LD C-Apochromat 40×/1.1 W objective.

### RhoA activity

For biochemically determination of RhoA activity after 5-HT_4_R stimulation, neurons were homogenized in lysis buffer (50 mM Tris-HCl, pH 8; 150 mM NaCl; 10 mM MgCl_2_; 1 mM EDTA; 1% TritonX-100) and centrifuged at 12,000 × *g* for 10 min at 4 °C. The cell extracts were incubated with an anti-active RhoA monoclonal antibody and protein A/G Agarose beads (New East Biosciences) for 1 h at 4 °C and then washed three times with lysis buffer. Active RhoA was analyzed by SDS-PAGE and subsequently immunoblotted with RhoA-specific antibody (67B9, Cell Signalling, 1:500).

### Antibodies used for western blots

Antibodies that were used for western blot analysis: anti G protein alpha S (1:500, Abcam); anti-Tubulin β-3 (1:1000, Covance); anti Cofilin (D3F9) XP (1:4000, Cell Signalling); anti-ERK (1:1000, Cell Signalling); anti GAPDH (Clone 6C5 AB2302, 1:10000, Millipore); anti Ga13 (A-20, sc-410, 1:500, Santa Cruz Biotechnology); Donkey anti-Goat IgG-HRP conjugate (1:20000, Santa Cruz Biotechnology), Goat anti-Rabbit IgG (H + L) HRP conjugate (1:10,000, Pierce); Rabbit anti-Goat IgG (H + L), HRP conjugate (1:10,000, Pierce); Rabbit anti-Mouse IgG Fc, HRP conjugate (1:10,000, Pierce).

### Imaging with a single-spine resolution

*RhoA activation and F-actin accumulation*. To determine spatiotemporal RhoA activity, N1E-115 cells or hippocampal neurons were transfected with the FRET-based RaichuEV-RhoA biosensor (YPet-PKN-RhoA-mTurquoise-KRas-CAAX). In addition, N1E-115 cells were co-transfected with 5-HT_4_R-mCherry or a pcDNA construct and neurons with a LifeAct-mRuby^34^ to visualize F-actin. Baseline RhoA activity was measured for 7.5 min. Afterward, 10 µM 5-HT or BIMU8 was added followed by incubation for 15 min. Images were acquired every 2.5 min in online fingerprinting mode using a Zeiss LSM 780 microscope with reference spectra YPet, mTurquoise and mCherry/mRuby. After live imaging, neurons were fixed and stained with anti-PSD-95 antibody. The acceptor/donor ratio (*R* = *F*_A_/*F*_D_) was used as biosensor readout. Offline analysis was done by custom-written Matlab scripts using the following scheme: image shift correction, correction for background, slight data blurring (kernel size 0.5), linear bleaching correction from time points before treatment for ratio images and pixel based ratio calculation.

*Two-photon (2P) excitation fluorescent imaging.* Organotypic hippocampal slices for 2P-excitation imaging were 7–14 DIV (2–9 days post-transfection). For the recordings, slices were transferred into a bicarbonate-buffered Ringer solution containing (in mM) 126 NaCl, 3 KCl, 2 MgSO_4_, 2 CaCl_2_, 26 NaHCO_3_, 1.25 NaH_2_PO_4_, 10 D-glucose, saturated with 95% O_2_ and 5% CO_2_ (pH 7.4; 300–310 mOsmol). Imaging was carried out with an Olympus FV1000 system optically linked a Ti:Sapphire MaiTai femtosecond-pulse laser (SpectraPhysics-Newport) at $\lambda _{\mathrm{ex}}^{2{\mathrm{P}}} = 860\,{\mathrm{nm}}$ (RhoA sensor optimum) or 820 nm with appropriate emission filters. Various digital zooms were used to collect images for high-resolution scanning (voxel size less than 0.08 × 0.08 × 0.5 µm^3^). For time-lapse monitoring of FRET-based RhoA sensor and LifeAct fluorescence, *z*-stacks of fluorescent images (typically 25–50 optical sections in 0.5-µm *z*-steps) were collected in a 2.5-min increment for 10 min before (baseline) and upon BIMU8 application (10 µM, overall 20–30 min recording). To avoid phototoxic damage to the tissue, the laser power was always kept below 4 mW. Recordings were made at 31–33 °C. Tissue scanned in the same way (the same time-frame mode) without agonist application (vehicle) was used as a control. In a separate set of experiments, organotypic hippocampal slices were pre-incubated with a high potent, specific ROCK inhibitor Y-27632 (50 µM for 1 h, 35 °C) as a control for RhoA inhibition.

### Electrophysiology

*Patch clamp recordings in dissociated neuronal cultures.* Whole-cell patch-clamp recordings were acquired in voltage-clamp mode using EPC-10/2 amplifier controlled by PatchMaster software (HEKA, Germany). The composition of the extracellular solution was as follows (in mM): 150 NaCl, 1 KCl, 2 CaCl_2_, 1 MgCl_2_, 10 HEPES, 10 glucose, 0.01 glycine, pH 7.3, osmolarity 320 mOsm. Gabazine (1 μM) and tetrodotoxin (TTX, 1 μM) were always present in the extracellular solution to block GABA_A_ receptors and sodium channels. The intracellular solution contained (in mM): 125 KmeSO_3_, 10 KCl, 5 Na_2_Phosphocreatine, 0.5 EGTA, 4 MgATP, 0.3 Na_2_GTP, 10 HEPES, pH 7.3, osmolarity 290 mOsm. Patch electrodes were pulled to reach the resistance of 3–6 MΩ. Postsynaptic current was low-pass filtered (2.9 kHz) and acquired at 20 kHz. Recordings with a leak current >200 pA at −70 mV or a series resistance of >50 MΩ were discarded. All recordings contain 5 mV voltage steps to track access resistance over time and correct current amplitude accordingly. mEPSCs were detected semi-automatically in Mini Analysis program with the same detection parameters and all traces were reviewed manually to correct for detection errors. All experiments were conducted at room temperature.

*Field potential recordings in situ*. Male and female 14–16-day-old C57BL6/J mice were killed by cervical dislocation, followed by decapitation. The brain was removed from the skull and transferred into an ice-cold artificial cerebrospinal fluid (ACSF), saturated with carbogen (95% O_2_, 5% CO_2_) and containing (in mM) 125 NaCl, 25 NaHCO_3_, 25 glucose, 2.5 KCl, 1.25 KH_2_PO_4_, 2 CaCl_2_, and 1.5 MgSO_4_ (pH 7.3). Both hippocampi were dissected out and sliced transversally (400 µm) using a tissue chopper with a cooled stage (custom-made by LIN, Magdeburg, Germany). Slices were kept at room temperature in carbogen-bubbled ACSF (95% O_2_ /5% CO_2_) for at least two hours before the start of recording. Recordings were performed in the same solution in a submerged chamber that was continuously superfused at 32 °C with carbogen-bubbled ACSF (1.2 ml/min). Recordings of field excitatory postsynaptic potentials (fEPSPs) were performed in the stratum radiatum of the CA1b subfield with a glass pipette filled with ACSF. The resistance of the pipette was 1 4 MΩ. Stimulation pulses were applied to Schaffer collaterals via a monopolar, electrolytically sharpened and lacquer-coated stainless steel electrode approximately 500 μm closer to the CA3 subfield than the recording electrode. Basal synaptic transmission was monitored at 0.05 Hz. LTP was induced by using theta burst stimulation (TBS), a pattern consisting of ten bursts applied at 5 Hz with each burst being composed of four pulses at 100 Hz and a width of the single pulses of 0.2 ms. The stimulation strength was set to provide baseline fEPSPs with slopes of approximately 50% of the subthreshold maximum. Short-term potentiation levels were measured as maximal levels of potentiation during first 5 min after induction of LTP. The data were recorded at a sampling rate of 10 kHz, then filtered (0–5 kHz) and analyzed using IntraCell software (custom-made, LIN Magdeburg, Germany). During the experiment, BIMU8 was bath-applied in ACSF at the concentration of 10 µM after 20 min of stable baseline recording for 60 min before TBS until the end of LTP recordings. For control experiments, the appropriate amount of bi-distilled water (vehicle) was applied.

*Whole-cell recordings in CA1 pyramidal neurons* *in situ*. Acute hippocampal slices (350 μm thick) were prepared from male Sprague-Dawley rats (P21–P24) in full compliance with the European Commission Directive (86/609/EEC) and the United Kingdom Home Office (Scientific Procedures) Act (1986). Transverse slices were cut in an ice-cold slicing solution containing (in mM) 64 NaCl, 2.5 KCl, 1.25 NaH_2_PO_4_, 0.5 CaCl_2_, 7 MgCl_2_, 25 NaHCO_3_,10 glucose and 120 sucrose (95% O_2_ and 5% CO_2_), then transferred into a Ringer solution where they were kept for at least 1 h before starting recordings. Whole-cell recordings were carried out either in current or voltage-clamp mode using a Multipatch 700B amplifier controlled by the pClamp 10.2 software (Molecular Devices, USA). Recordings of miniature and evoked excitatory postsynaptic currents (EPSC) were made from CA1 pyramidal neurons at −70 mV in a Ringer solution in the presence of picrotoxin (200 μM) and CGP-52432 (3 μM) at 31–33 °C (including also 1 μM TTX when recording mEPSCs). The recording electrodes had a resistance 2.5–4.5 MΩ when filled with the intracellular solution containing (in mM) 135 K-methanesulfonate, 10 Tris-phosphocreatine, 4 MgCl_2_, 0.2 EGTA, 10 HEPES, 4 Mg-ATP, 0.4 GTP-Na (pH 7.2, osmolarity ~290 mOsm). For recordings of evoked EPSCs, 5 mM QX-314 was also added to the intracellular solution. To stimulate the bulk of Schaffer collaterals, a bipolar stimulating electrode was placed in stratum radiatum ~200 μm from stratum pyramidale. The stimulus was 20–300 μA intensity and of 100-μs duration; the protocol consisted of 5 pulses applied at 20 Hz pulse train to evoke EPSCs at a low-frequency stimulation mode. Recordings (500 ms-duration sweeps) were filtered and digitized; individual sweeps were collected at a 20-s interval.

The cell access resistance was normally settled within 5–7 min post break-in and was, in baseline conditions, 15 ± 1 MΩ (mean ± SEM) in the beginning, increasing slightly to 17 ± 2 MΩ over ~45 min of recording. Under BIMU8 application, these values were 16 ± 2 MΩ to begin with, and 15 ± 1 MΩ in 50 min of recording. During the experiment, this value could fluctuate by up to ±30% to reflect changes in the holding current induced by the agonist.

*Statistics and reproducibility*. Statistical analysis was done using GraphPad Prism7 software. Quantitative analysis of the western blots was done by the sum of replicates^83^. Student’s *t*-test (two-tailed paired or unpaired) was used to determine the statistical difference between experimental groups. Analysis of variance (ANOVA) followed by post-hoc tests was used for multiple comparisons (one-way or two-way where appropriate). A *p*-value of <0.05 was considered as statistically significant for either test. Data are represented as mean value ± SEM.

When the same plasmid or genotype were analyzed after different treatments, one-way ANOVA test was used, when different plasmids were treated with the same agonist/antagonist two-way ANOVA was used with indicated post-hoc tests. The control condition was set to one. In microscope experiments on N1E-115 cell, each condition contained at least five to ten cells per experiment. In experiments monitoring morphology of dendritic spines, each condition contained at least 5–30 cells (at least 156–435 individual dendritic spines) if not indicated differently. We performed nested-ANOVA analysis and then Newman–Keuls Multiple Comparison Post Test for experiments monitoring short-term changes in morphology of dendritic spines in dissociated neuronal culture. Here, cells were used as statistical unit. For statistical analysis, the means of all treatment conditions within one group and the KO and WT or ctrl condition were compared to each other.

### Reporting summary

Further information on research design is available in the Nature Research Reporting Summary linked to this article.

## Supplementary information

Supplementary Information

Supplementary Movie 1

Supplementary Movie 2

Supplementary Movie 3

Supplementary Movie 4

Supplementary Movie 5

Description of Additional Supplementary Files

Supplementary Data 1

Reporting Summary

## Data Availability

All data supporting the findings of this study are available within the article and its Supplementary Information file or are available from the corresponding author upon reasonable request.
