# Supplementary material for: Serotonin 5-HT4 receptor boosts functional maturation of dendritic spines via RhoA-dependent control of F-actin
Source: Commun Biol. 2020 Feb 14;3:76. doi: 10.1038/s42003-020-0791-x (PMC7021812; doi:10.1038/s42003-020-0791-x)
Supplement: Supplementary file 1 — Supplementary Information [file 42003_2020_791_MOESM1_ESM.pdf]

SUPPLEMENTARY INFORMATION

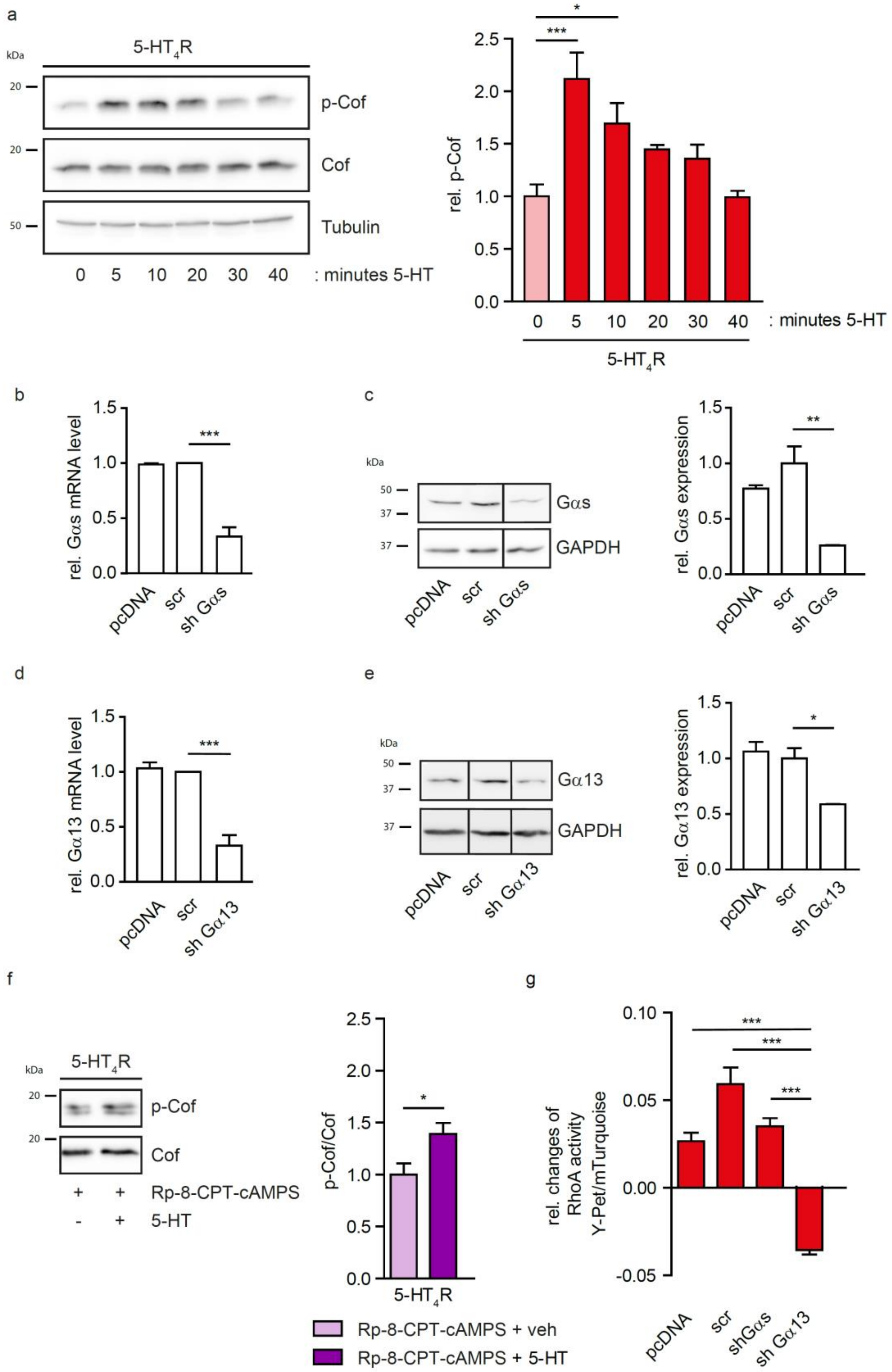

Supplementary Fig. 1. Time kinetics for cofilin phosphorylation and validation of shRNA against Gαs and Gα13 (related to Fig. 1).

a. (Left) Representative western blot of the 5-HT_4R mediated cofilin phosphorylation kinetics in N1E-115 cells transfected with 5-HT_4R-eGFP and treated with 5-HT for different times as indicated on the bottom. (Right) Quantification of the relative cofilin phosphorylation calculated as the ratio of p-Cof to total Cof normalized to Tubulin expression (n = 4; *p < 0.05, ***p < 0.001, one-way ANOVA with Dunnett test).

b – e. N1E-115 cells were transfected with shRNAs as indicated and both amount of the mRNA encoding Gαs (**b**) or Gα13 (**d**) and protein expression levels (**c** and **e**) were analyzed by qRT-PCR and western blots, respectively (right). Cells transfected with scrambled (scr) shRNA or pcDNA were used as a control (n = 3). *$P < 0.05$, **$P < 0.01$, ***$P < 0.001$ (**b** and **d**) one-way ANOVA with Dunnett test. Data are represented as mean + SEM.

f. Representative western blot (left) and quantification (right) of the 5-HT_4R-mediated cofilin phosphorylation in N1E-115 cells transfected with 5-HT_4R-eGFP and pretreated with PKA inhibitor Rp-8-CPT-cAMPS. *$P < 0.05$

g. Relative changes in 5-HT_4R-mediated RhoA activity in N1E cells co-expressing 5-HT4R and FRET-based biosensor Raichu-RhoA along with indicated constructs.

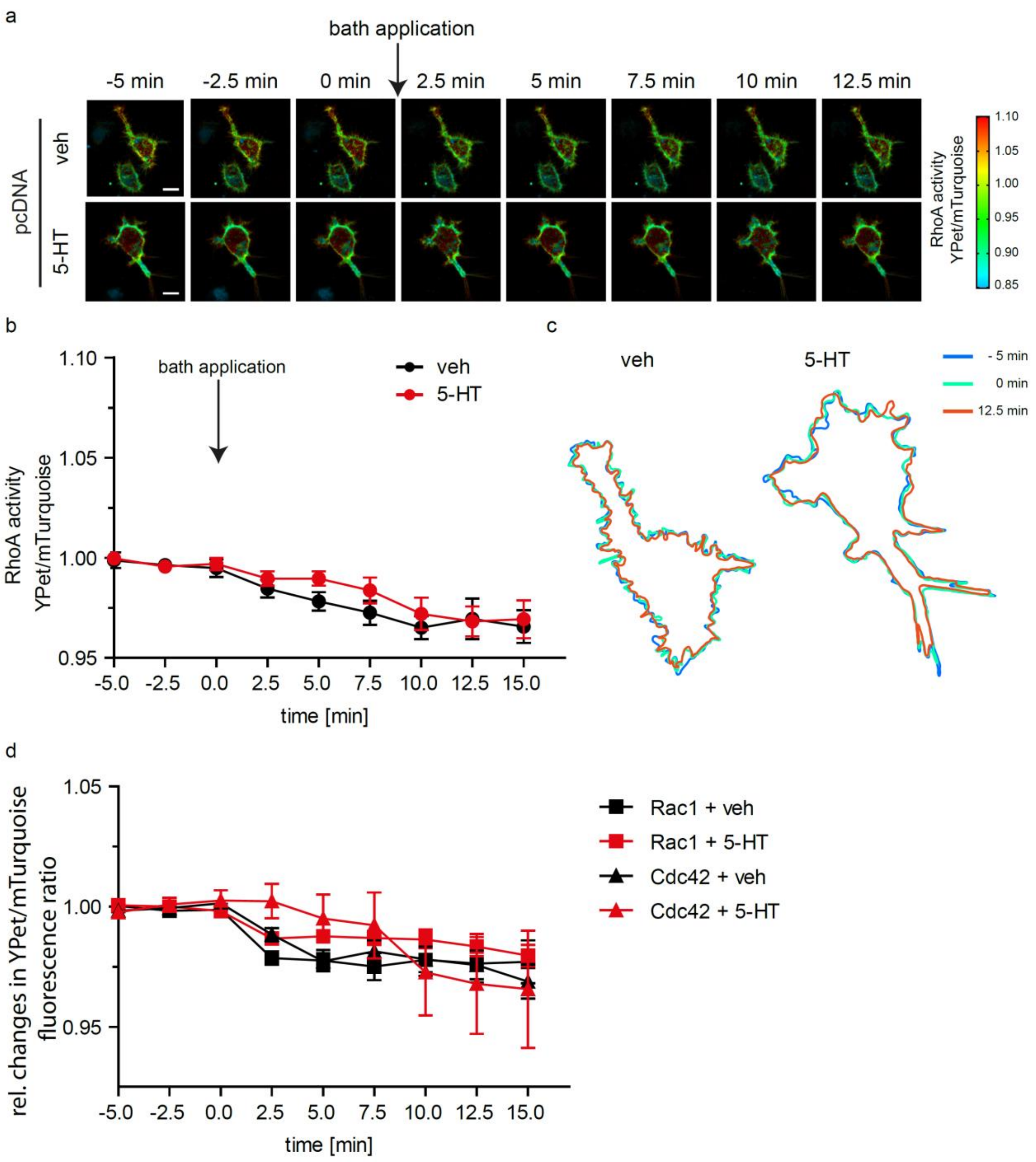

Supplementary Fig. 2. RhoA activity in N1E cells in absence of 5-HT$_4$R and impact of 5-HT on morphology of N1E cells expressing 5-HT$_4$R (related to Figs. 1 and 2).

a. Representative time-lapse confocal images of N1E-115 cells co-transfected with pcDNA and FRET-based RhoA-biosensor and treated with vehicle (veh) or 5-HT. Images are color-coded for the YPet/mTurquoise ratio. Scale bar, 20 µm.

b. Quantification of RhoA activity (YPet/mTurquoise ratio; mean ± SEM) in N1E-115 cells over time after treatment with veh or 5-HT (n = 3 with at least 6 cells analyzed per condition; no significant differences between veh and 5-HT, Mann-Whitney test).

c. Morphology contour of N1E-115 cells treated with veh or 5-HT at -5 min, 0 min and 12.5 min.

d. Quantification of Rac1 and Cdc42 activity (YPet/mTurquoise ratio; mean ± SEM) in N1E-115 cells over time after treatment with veh or 5-HT no significant differences between veh and 5-HT for both small GTPases were obtained.

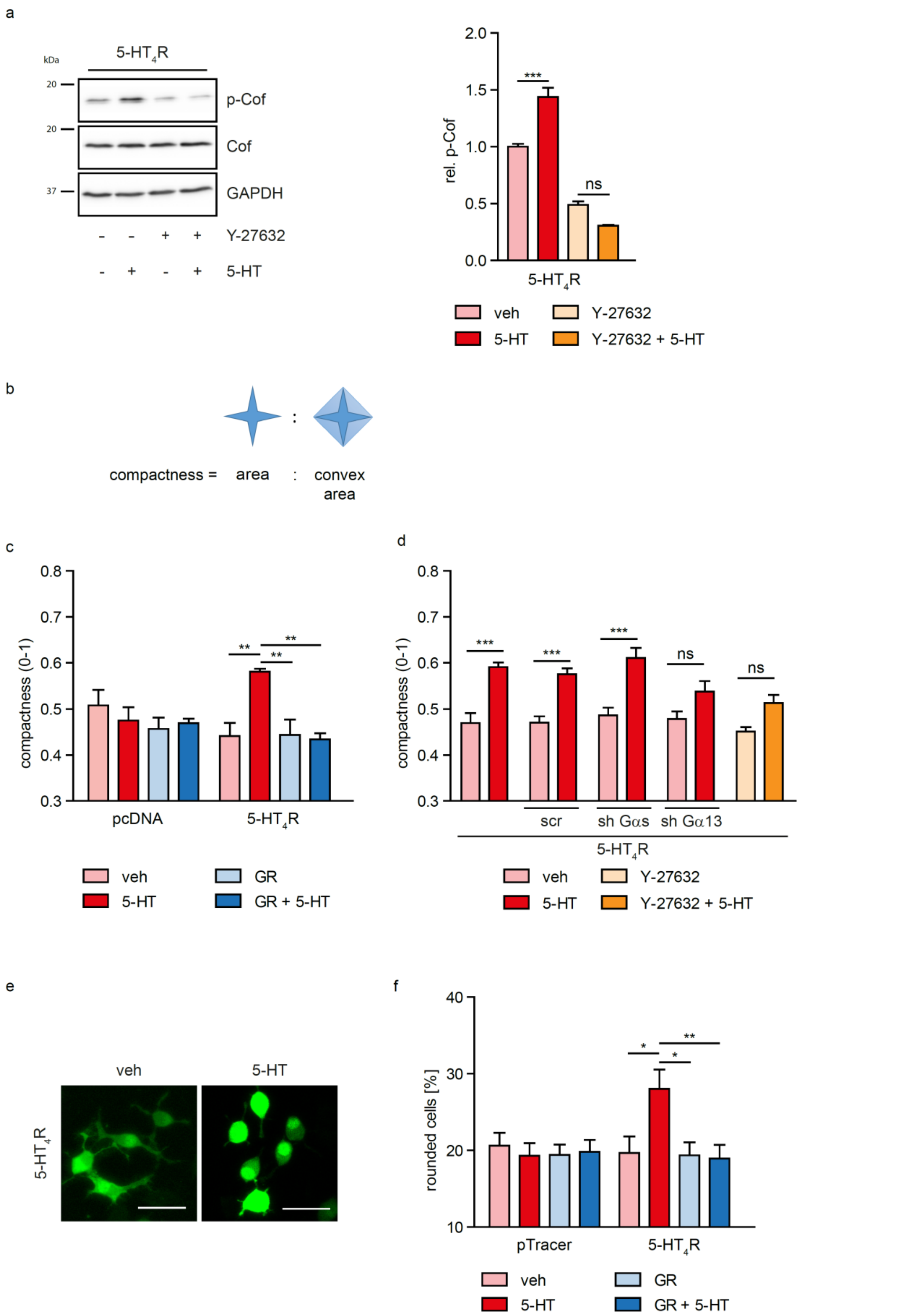

Supplementary Fig. 3. Impact of 5-HT on morphology of N1E cells expressing 5-HT4R (related to Figs. 1 and 2).

a. N1E-115 cells transfected with pcDNA or 5-HT_4R-eGFP were treated for 5 minutes with 5-HT, 5-HT_4R agonist BIMU8 alone or after pre-incubation with a high potent, ROCK inhibitor Y-27632 (50 µM), followed by the western blot analysis with antibodies against phosphorylated cofilin (upper row, p-Cof), total cofilin (middle row, Cof) and GAPDH (bottom*).*Quantification of the relative cofilin phosphorylation is shown on the right.

b. Graphical definition of parameter "compactness"

c. Analysis of compactness in N1E-115 cells expressing 5-HT_4R, calculated as shown in (**b**). Impact of 5-HT on morphology of N1E-115 cells expressing 5-HT_4R (n=4 with at least 8 cells analyzed per condition/experiment). **P < 0.01, two-way ANOVA with Tukey test)

d. Analysis of compactness after Gαs and Gα13 knockdown with specific shRNAs or after pre-incubation with a high potent, specific ROCK inhibitor Y-27632 (50 µM) (n=6 with at least 7 cells analyzed per condition per experiment; ***p < 0.001, two-way ANOVA with Sidak test)

e. Representative images of N1E-115 cells transfected with pTracer-5-HT_4R after 90 min treatment with vehicle or 5-HT. Scale bar, 50 µm.

f. Morphometric analysis of N1E-115 cells transfected with pTracer-5-HT_4R and treated with vehicle (veh), 5-HT or 5-HT together with GR113808 for 90 minutes (n=4, between 100 and 400 cells were analyzed per condition/experiment; *p < 0.05, **p < 0.01, two-way ANOVA with Tukey test).

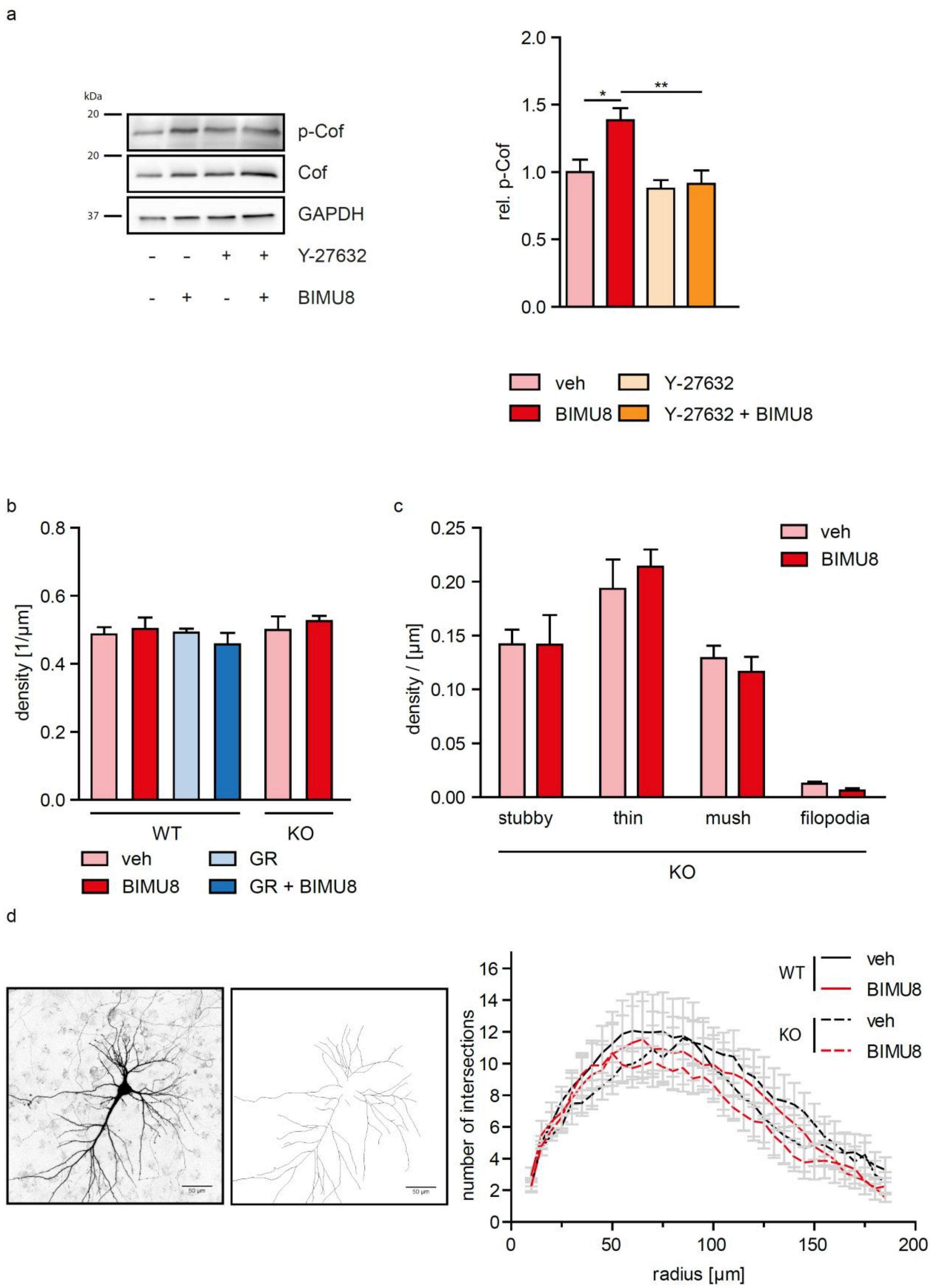

Supplementary Fig. 4. Cofilin phosphorylation, spine morphology, spine density and dendritic arborization in neurons after 5-HT4R activation (related to Fig. 3).

a. Hippocampal neurons (DIV12) isolated from WT mice were treated with the 5-HT4R agonist BIMU8 with or without pre-treatment with specific ROCK inhibitor Y-27632 (50

µM), followed by the western blot with antibodies against phosphorylated (upper row, p-Cof), total cofilin (middle, Cof) and GAPDH as a loading control (bottom). (Left) Representative western blot showing cofilin phosphorylation. (Right) Quantification of the relative cofilin phosphorylation. *p < 0.05, **p < 0.01.

b. Analysis of spine density in hippocampal neurons (DIV12) from WT and 5-HT$_4$R KO mice treated with vehicle (veh), BIMU8 or GR for four days (n = 4, one-way ANOVA with Tukey test).

c. Quantification of the density of stubby spines, thin spines, mushroom (mush) spines, and filopodia in hippocampal neurons from 5-HT$_4$R KO (n = 4, unpaired t-test).

d. Representative hippocampal neuron at DIV12 (left) and the marked dendrites (middle), which were subjected to the Sholl analysis. Scale bar, 50 µm (Right) Analysis of dendritic branching in WT and 5-HT$_4$R KO (n = 3; one-way ANOVA).

All data are shown as mean + SEM.

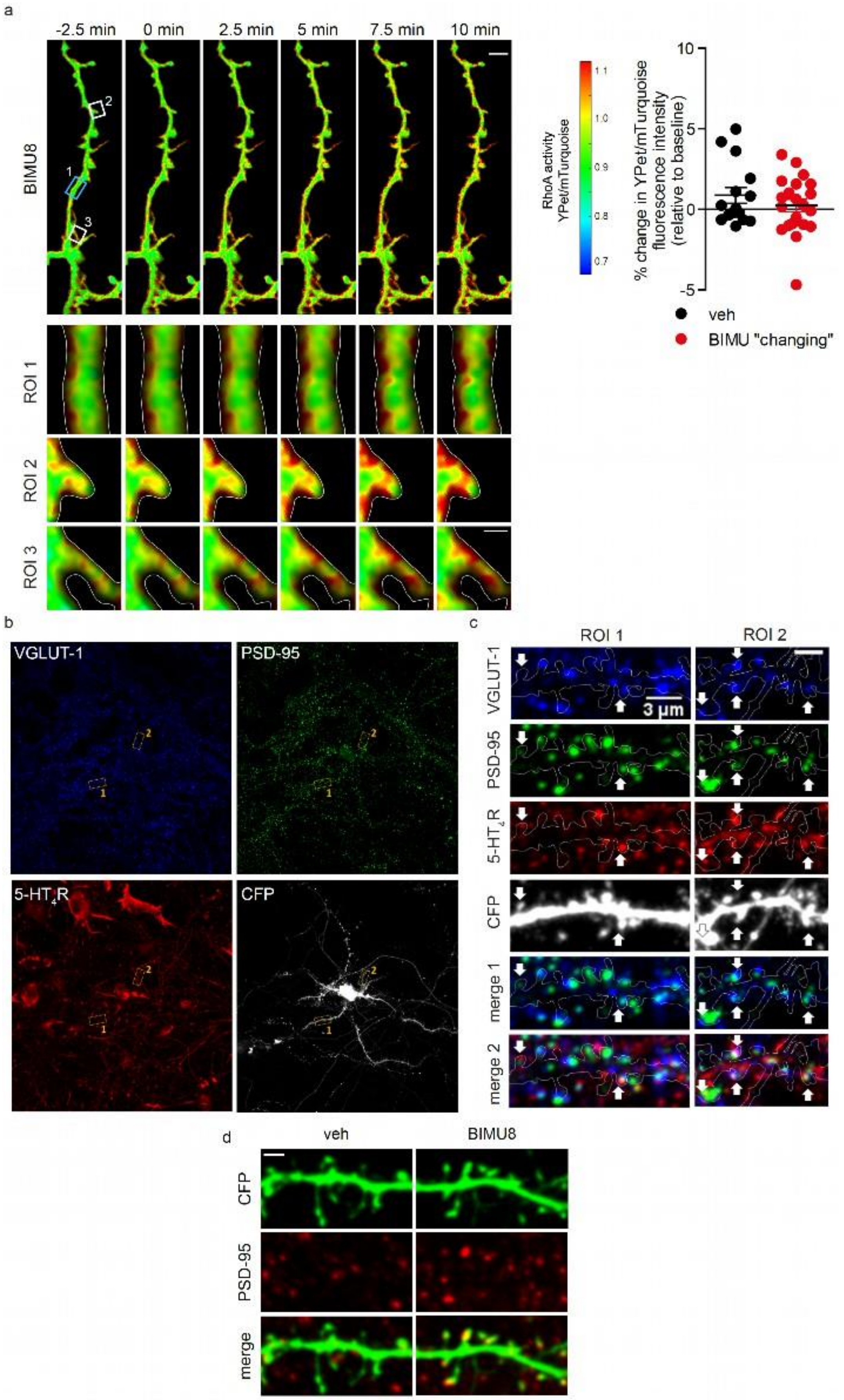

Supplementary Fig. 5. RhoA activity, synaptic expression of 5-HT$_4$R and the enhanced PSD-95 immunostaining after 5-HT$_4$R activation (related to Fig. 4).

a. Representative time-lapse confocal images of dendritic shaftes (top left) in the cerulean-expressing hippocampal neurons co-transfected with FRET-based biosensor Raichu-RhoA. Images were acquired every 2.5 min. BIMU8 was added to the bath solution at time point 0 and cells were imaged for the further 10 min. Fluorescence intensity for ratiometric changes in the YPet/mTurquoise ratio, reflecting the RhoA activation, is shown. Upper row: Overview image without correction for bleaching. Three bottom rows: Enlargement of 3 regions of interest (ROIs) depicted in the upper image. ROI1 shows a part of dendritic shaft, Rois2 and 3 show single spines. Each ROI was separately corrected for bleaching. (Right) Quantification of the YPet/mTurquoise fluorescence intensity ratio in dendritic shafts in control and BIMU8 responding spines.

b – c. Representative images of CFP-transfected hippocampal WT neurons (white) immunostained with antibodies to visualize VGLUT-1 (blue), PSD-95 (green) and 5-HT_4R (red) (**b**), with the enlarged regions of interest (i.e., ROI1 and ROI2) in (**c**). Two bottom rows in (**c**) show colocalization between synaptic markers VGLUT-1 and PSD-95 “merge 1” and between synaptic markers (VGLUT-1, PSD-95) and 5-HT_4R (“merge 2”).

d. Representative images of CFP-transfected hippocampal neurons (green) treated with vehicle (veh) or BIMU8 for 10 min followed by immunostaining with antibody against PSD-95 (red). Note the increased PSD-95 immunostaining in a BIMU8-treated neuron. Scale bar: 2 µm.

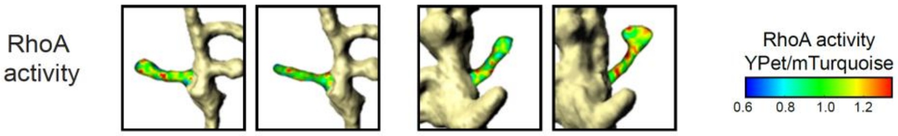

Supplementary Fig. 6. 5-HT$_4$R/RhoA signaling mediates dendritic spine maturation in the hippocampal tissue (related to Fig. 5).

(Upper panel) The time-lapse 2P excitation images of dendritic spines in hippocampal neurons transfected with FRET-based biosensor Raichu-RhoA acquired as Z-stacks every 2.5 min (typically 20 to 50 optical sections; 512 × 512 pixel frames, 0.5 μm Z-steps, voxel size less than 0.08 μm^3). After 7.5 min imaging for baseline (RhoA activity under control conditions, -7.5 min to 0 min), either vehicle or BIMU8 were added to the bath solution and the same region of interest was scanned for the next 20 min. Images show the time-course of changes in the RhoA activity within defined spines (color-coded, as indicated on the bottom). (Lower panel) Quantification of the YPet/mTurquoise fluorescence intensity ratio in control and BIMU8 responding spines. $^*p < 0.05$.

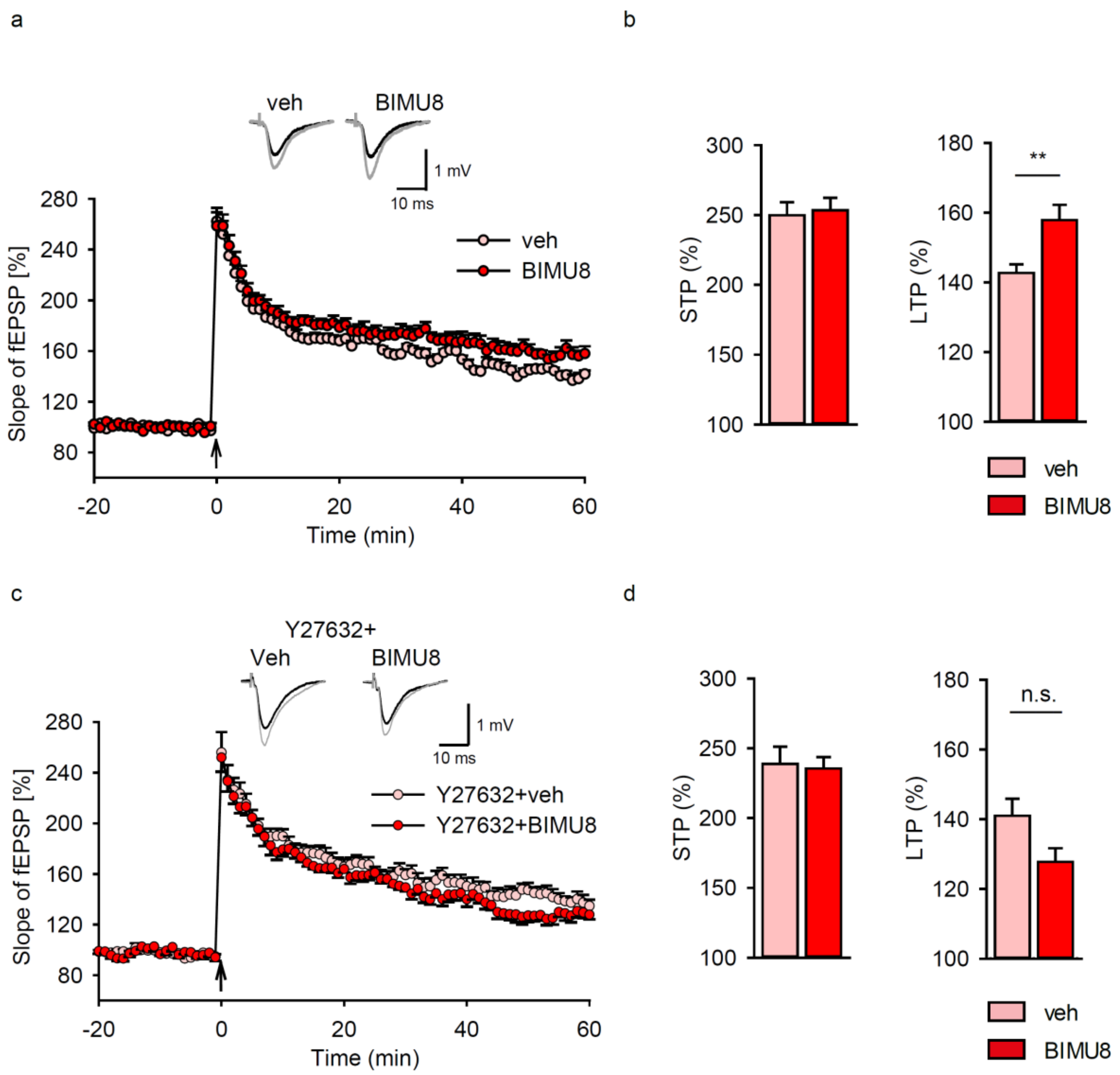

Supplementary Fig. 7. Activation of 5-HT$_4$Rs leads to the enhanced LTP in the CA3 - CA1 circuits (related to Fig. 6).

a and **c**. Representative traces and changes in the slope of fEPSPs recorded in the stratum radiatum in response to stimulation of Shaffer collaterals in acute hippocampal slices from P14 mice before (black) and 1 hour after (grey) induction of long-term potentiation (LTP, theta-burst stimulation, indicated by arrow, time 0) in control (veh) and BIMU8-treated slices without (**a**) and with (**c**) pre-treatment with the specific ROCK inhibitor Y27632.

b and **d**. Summary of STP and LTP levels at 0-2 min and 50-60 min, respectively, after theta-burst stimulation in slices pre-treated without (**b**) and with (**d**) the specific ROCK inhibitor Y27632 (veh: n = 13 slices; BIMU8: n=10; Y27632-veh: n=8; Y27632-BIMU8: n=8; slices are from at least 5 mice in each group; **$p < 0.01$, two-tailed t-test). Data show relative changes to the mean initial slope of fEPSPs recorded 20 min before LTP induction.

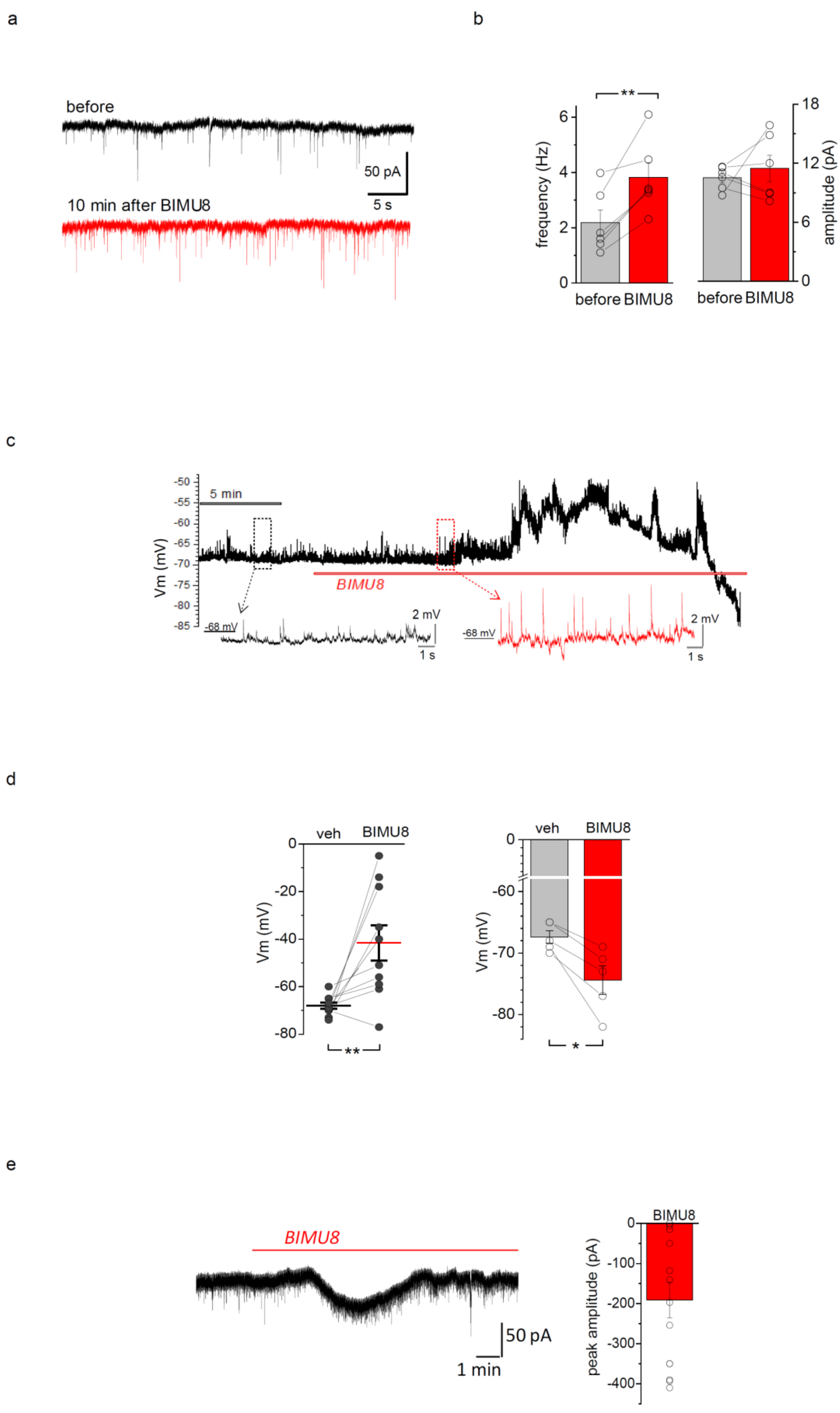

Supplementary Fig. 8. Activation of 5-HT4Rs modulates intrinsic excitability of CA1 pyramidal neurons (related to Fig. 6).

a. Representative traces of miniature EPSCs recorded in a CA1 pyramidal neuron before and 10 min after BIMU8 application (10 µM, bath application in the presence of TTX, picrotoxin and CGP-52432).

b. Summary of the miniature EPSC frequency and amplitude recorded in CA1 neurons before (for the 10 min time period) and after BIMU treatment (n = 8242 events for baseline and n = 12751 events recorded in 6 neurons after BIMU8 application; *p < 0.01, two-tailed paired t-test).

c. Example trace, current-clamp whole-cell recording the membrane potential (V_m) in a CA1 pyramidal neuron, before and after activation of 5-HT_4R with BIMU8 (10 µM, bath application); boxes, areas shown on the expanded scale below, as indicated.

d. Statistical summary of experiments shown in C; peak magnitude of membrane depolarization (n = 10 cells, left plots), followed by a membrane hyperpolarization (n = 5 out of 10 cells, right). *p < 0.05, **p < 0.01, paired *t*-test).

e. Example trace of the BIMU8-induced current in a CA1 pyramidal neuron at –70 mV (left) and summary of the current amplitude in CA1 neurons (right) elicited by activation of 5-HT_4R with BIMU8 (10 µM, bath application; n = 10 cells). Data are mean ± SEM.

Supplementary Fig. 9. (Raw data for the western blots included in each main figure)

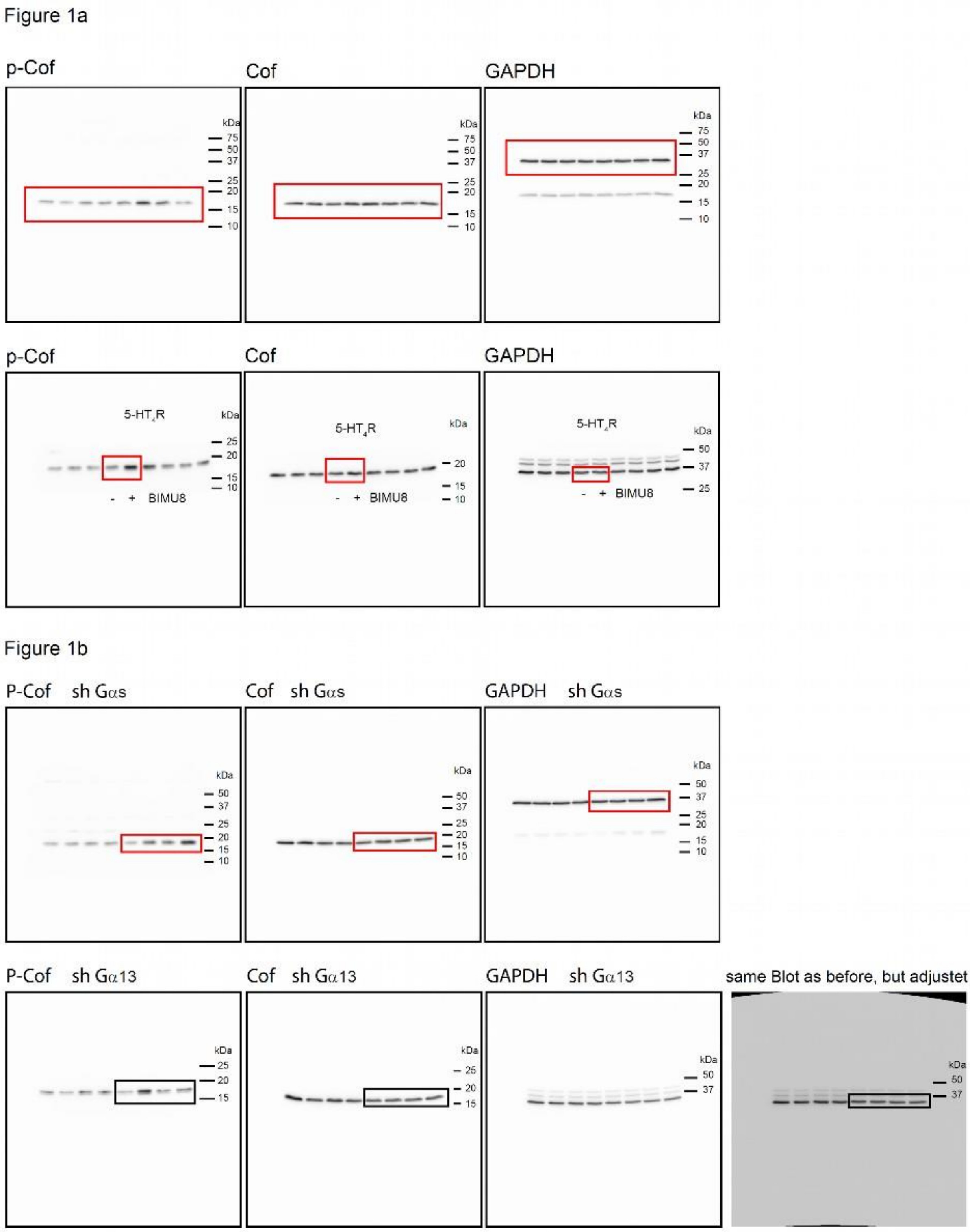

Figure 1c

p-Cof Cof GAPDH

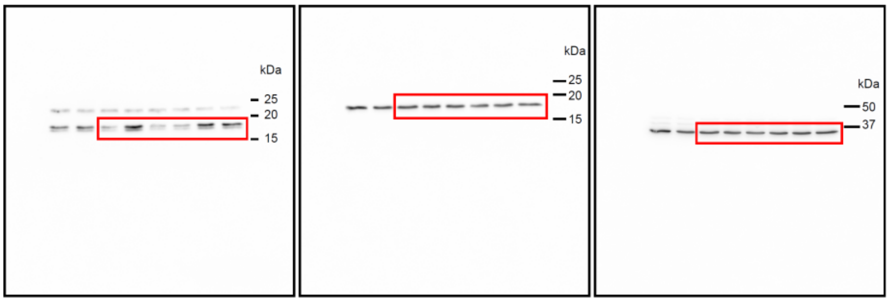

Figure 2a

actin

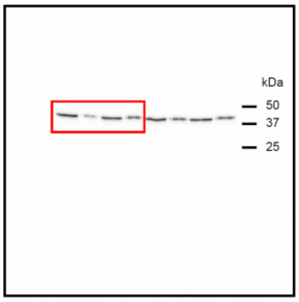

Figure 3a

p-Cof Cof GAPDH

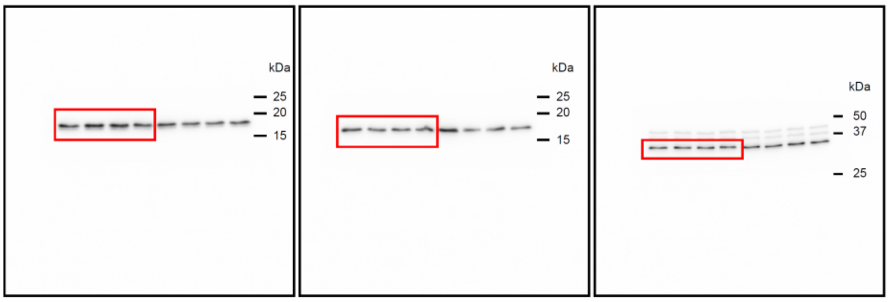

Figure 3b

p-Cof Cof GAPDH

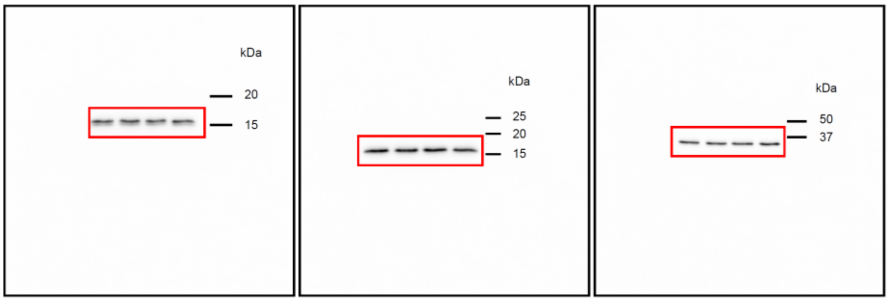

Figure 4a

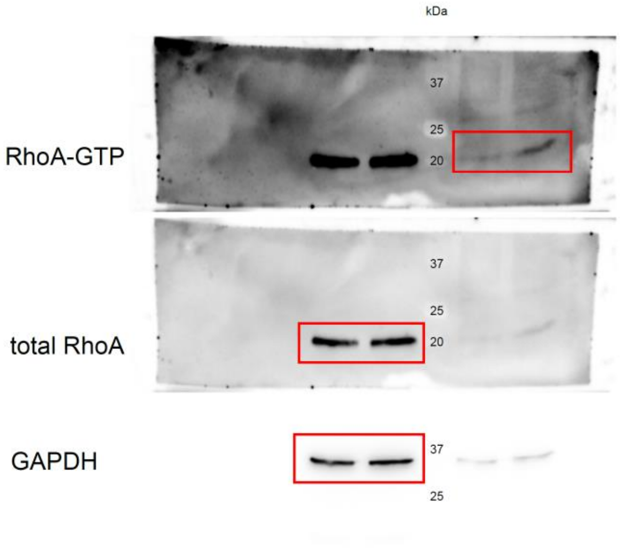

165
