# Supplementary material for: Serotonin 5-HT4 receptor boosts functional maturation of dendritic spines via RhoA-dependent control of F-actin
Source: Commun Biol. 2020 Feb 14;3:76. doi: 10.1038/s42003-020-0791-x (PMC7021812; doi:10.1038/s42003-020-0791-x)
Supplement: Supplementary file 7 — Description of Additional Supplementary Files [file 42003_2020_791_MOESM7_ESM.pdf]

Description of Additional Supplementary Files

File Name: **Supplementary Movie 1**

Description: Time-lapse confocal ratiometric images of the single N1E-115 cells co-transfected with 5-HT_4R together with FRET-based biosensor Raichu-RhoA. After 5 min imaging under control conditions, 5-HT was added to the bath solution (indicated by the red spot) and cells were imaged for the next 15 min. Increase in the YPet/mTurquoise ratio is equivalent to an increase in the RhoA activity.

File Name: **Supplementary Movie 2**

Description: Time-lapse confocal ratiometric images of one filopodia from N1E-115 cells cotransfected with 5-HT_4R together with FRET-based biosensor Raichu-RhoA. After 5 min imaging under control conditions, 5-HT was added to the bath solution (indicated by the red spot) and cells were imaged for the next 15 min. Increase in the YPet/mTurquoise ratio is equivalent to an increase in the RhoA activity.

File Name: **Supplementary Movie 3**

Description: Time-lapse confocal images of defined spines in the hippocampal neurons cotransfected with FRET-based biosensor RaichuRhoA (left) and LifeAct-mRuby (right). Images were acquired every 2.5 min. After 7.5 min imaging under control conditions (-7.5 min to 0 min), BIMU8 was added to the bath solution and cells were imaged for the further 10 min. Increase in the YPet/mTurquoise ratio is equivalent to an increase in the RhoA activity in a single spine. Increased fluorescence intensity for LifeActmRuby indicates the F-actin accumulation in the same spines.

File Name: **Supplementary Movie 4**

Description: 3D reconstruction of dendrites in hippocampal neurons transfected with FRET-based biosensor Raichu-RhoA acquired before and 15 min after treatment with vehicle. Images also show the RhoA activity within the defined spine (color-coded, as indica ted on the bottom).

File Name: **Supplementary Movie 5**

Description: 3D reconstruction of dendrites in hippocampal neurons transfected with FRET-based biosensor Raichu-RhoA acquired before and 15 min after treatment with BIMU8. Images show also the RhoA activity within the defined spine (color-coded, as indica ted on the bottom).

File Name: **Supplementary Data 1**

Description: Table containing all source data underlying the graphs and charts presented in the main figures.
