# Supplementary material for: Serotonin 5-HT4 receptor boosts functional maturation of dendritic spines via RhoA-dependent control of F-actin
Source: Commun Biol. 2020 Feb 14;3:76. doi: 10.1038/s42003-020-0791-x (PMC7021812; doi:10.1038/s42003-020-0791-x)
Supplement: Supplementary file 9 — Reporting Summary [file 42003_2020_791_MOESM9_ESM.pdf]

nature research

Corresponding author(s): Evgeni Ponimaskin

Last updated by author(s): Jan 9, 2020

Reporting Summary

Nature Research wishes to improve the reproducibility of the work that we publish. This form provides structure for consistency and transparency in reporting. For further information on Nature Research policies, see Authors & Referees and the Editorial Policy Checklist.

Statistics

For all statistical analyses, confirm that the following items are present in the figure legend, table legend, main text, or Methods section.

n/a	Confirmed	
☐	☒	The exact sample size (n) for each experimental group/condition, given as a discrete number and unit of measurement
☐	☒	A statement on whether measurements were taken from distinct samples or whether the same sample was measured repeatedly
☐	☒	The statistical test(s) used AND whether they are one- or two-sided<br>*Only common tests should be described solely by name; describe more complex techniques in the Methods section.*
☒	☐	A description of all covariates tested
☐	☒	A description of any assumptions or corrections, such as tests of normality and adjustment for multiple comparisons
☐	☒	A full description of the statistical parameters including central tendency (e.g. means) or other basic estimates (e.g. regression coefficient) AND variation (e.g. standard deviation) or associated estimates of uncertainty (e.g. confidence intervals)
☒	☐	For null hypothesis testing, the test statistic (e.g. F, t, r) with confidence intervals, effect sizes, degrees of freedom and P value noted<br>*Give P values as exact values whenever suitable.*
☒	☐	For Bayesian analysis, information on the choice of priors and Markov chain Monte Carlo settings
☒	☐	For hierarchical and complex designs, identification of the appropriate level for tests and full reporting of outcomes
☒	☐	Estimates of effect sizes (e.g. Cohen's d, Pearson's r), indicating how they were calculated

Our web collection on statistics for biologists contains articles on many of the points above.

Software and code

Policy information about availability of computer code

Data collection	IntraCell software (custom-made, LIN Magdeburg, Germany) was used for data collection by LTP measurements
Data analysis	- OriginPro 2018 and pClamp10 software (Molecular Devices) were used for analysis and recordings of electrophysiological data in organ otypic preparations. - Custom written Matlab scripts were used for preprocessing and analysis of FRET biosensors data and F- and G-acti n results.<br>- ImageJ was used for analysis of fluorescence intensity of images obtained by confocal microscopy<br>- Spine Magick (patent no. WO/2013/021001) was used for spine analysis in cultured neurons.<br>- 3DSpAn and Imaris were used for 3D visualition of dendritic spines from organotypic culture.<br>- SigmaPlot 12.0 and GraphPad Prizm8 were used for statistical data analysis.

For manuscripts utilizing custom algorithms or software that are central to the research but not yet described in published literature, software must be made available to editors/reviewers. We strongly encourage code deposition in a community repository (e.g. GitHub). See the Nature Research guidelines for submitting code & software for further information.

Data

Policy information about availability of data

All manuscripts must include a data availability statement. This statement should provide the following information, where applicable:

- Accession codes, unique identifiers, or web links for publicly available datasets
- A list of figures that have associated raw data
- A description of any restrictions on data availability

The datasets generated during and/or analysed during the current study are available from the corresponding author on reasonable request

Field-specific reporting

Please select the one below that is the best fit for your research. If you are not sure, read the appropriate sections before making your selection.

☒ Life sciences ☐ Behavioural & social sciences ☐ Ecological, evolutionary & environmental sciences

For a reference copy of the document with all sections, see nature.com/documents/nr-reporting-summary-flat.pdf

Life sciences study design

All studies must disclose on these points even when the disclosure is negative.

Sample size

No predetermine sample-size calculation was performed; all sample sizes were based on statistically significant difference between experimental groups. Sample size was selected according to previous experience and publications.

Data exclusions

gs

In general, no data were excluded from the analyses.
During the LTP measurements, several slices were excluded independently of LTP values, only if a technical problem appeared during recordin

(dust of recording electrode, insufficient solution oxygenation), which manifested as sudden changes in fEPSP amplitude. All such cases are properly documented in the lab book.
In addition, Western blots with air bubbles affecting quantification were excluded from analysis.

Replication

All experimental findings showed the reproducibility and were replicated through the repeated experiments.
In case of LTP, no extra-replication was done as this is quite reliable assay used for many years in the lab of Dr. Dityatev

Randomization

In general, all samples were allocated in a random way. Tissue samples were also randomly allocated into experimental groups for both organotypic and acute slice preparations in electrophysiological and 2P florescence imaging experiments.
In case of LTP measurement, slices from the same mouse were randomly attributed to control or treatment groups.

Blinding

-The analysis of the spines morphology, N1E-115 cell morphology, F/G-actin ratio were done blindly. The analysis of biosensors were done by Matlab script automatically and ROI selection was done blindly.
- Blinding was not relevant to the electrophysiological data of neuronal excitability/synaptic transmission where agonists were acutely applied to tissue preparations or neuronal cultures (i.e. before-after treatment).
- In case of LTP measurements, no blinding was performed as this methodological approach is robust and there are no subjective decisions du ring data
acquisition and analysis.
- In case of Western blot, blinding was not relevant because the method used is robust and there are no subjective decisions during data acquisition and analysis

Reporting for specific materials, systems and methods

We require information from authors about some types of materials, experimental systems and methods used in many studies. Here, indicate whether each material, system or method listed is relevant to your study. If you are not sure if a list item applies to your research, read the appropriate section before selecting a response.

Materials & experimental systems

n/a	Involved in the study	
☐	☒	Antibodies
☐	☒	Eukaryotic cell lines
☒	☐	Palaeontology
☐	☒	Animals and other organisms
☒	☐	Human research participants
☒	☐	Clinical data

Methods

n/a	Involved in the study	
☒	☐	ChIP-seq
☒	☐	Flow cytometry
☒	☐	MRI-based neuroimaging

Antibodies

Antibodies used

Active RhoA (26904) , mouse, monoclonal, 1:1000, New East Biosciences
RhoA (67B9), rabbit, monoclonal, 1:1000, Cell Signaling, lot nr 0005
PSD-95 (7E3-1B8), mouse, monoclonal, 1:400, Thermo Fisher Scientific, lot nr ME153286
VGLUT-1 (135 304), guinea pig, polyclonal, 1:1000, Synaptic Systems
5-HT4R (ASR-036), rabbit, polyclonal, 1:200, Alomone, lot nr ASR036AN0102
Anti-Tubulin ß-3, goat, polyclonal, 1:1000, Covance
Cofilin (D3F9) XP, rabbit, polyclonal, 1:4000, Cell Signaling
P-cofilin (hSer3), rabbit, polyclonal, 1:4000, Santa Cruz Biotechnology
GAPDH (Clon 6C5) (AB2302), mouse, polyclonal, 1:10000; Millipore
Anti G protein alpha S, goat, polyclonal, 1:500, Abcam

	Galpha13 (A-20)sc-410, rabbit, polyclonal, 1:500, Santa Cruz Biotechnology<br>beta-Actin (A2066), rabbit, polyclonal, 1:1000, Sigma<br>Donkey anti-Goat IgG-HRP conjugate, 1:20000, Santa Cruz Biotechnology (anfangs)<br>Goat anti-Rabbit IgG (H+L), HRP conjugate, 1:10000, Pierce<br>Rabbit anti-Goat IgG (H+L), HRP conjugate, 1:10000, Pierce<br>Rabbit anti-Mouse IgG Fc, HRP conjugate, 1:10000, Pierce
Validation	Blocking peptide provided by the manufacturer was used to validate the 5-HT_4R antibody.<br>All other primary antibodies were confirmed by manufacturer to be specific and were applied in multiple published studies.

Eukaryotic cell lines

Policy information about cell lines

Cell line source(s)	Mouse neuroblastoma N1E-115 cell line was purchased by ATCC (ATCC® CRL-2263™)
Authentication	N1E-115 cell line was authenticated by ATCC
Mycoplasma contamination	N1E-115 cell line was micoplasma negative as verified by micoplasma test performed by Eurofins Company (former GATC Biotech)
Commonly misidentified lines<br>(See ICLAC register)	No misidentified cell lines were used in the study

Animals and other organisms

Policy information about studies involving animals; ARRIVE guidelines recommended for reporting animal research

Laboratory animals	- Male Sprague-Dawley rats (P21-P24) and Sprague-Dawley rat pups (P6–P8) (organotypic preparations)<br>- Male and female 14- to 16-day-old C57BL6/J mice (LTP)<br>- Pups (P1) of C57BL/6J mice were used for preparation of primary neuronal cultures<br>-The generation of 5-HT_4R KO and WT mice on a C57BL/6J background from heterozygous breeding has been described and engineered previously by V. Compan [(Compan et al., J. Neurosci. 2004), MGI n°3027491].
Wild animals	The study did not involve wild animals
Field-collected samples	The study did not involve samples collected from the field
Ethics oversight	All procedures performed on animals were according to the guidelines of the European Commission (European Communities Council Directive 2010/63/EU) and the United Kingdom Home Office (Scientific Procedures) Act (1986).<br>Experiments were approved by the local animal care committee (Landesverwaltungsamt Sachsen-Anhalt).

Note that full information on the approval of the study protocol must also be provided in the manuscript.
